# Sex-Based Differences in Objective Measures of Quadriceps Recovery Following Anterior Cruciate Ligament Reconstruction in Adults: A Systematic Review

**DOI:** 10.3390/sports14100432

**Published:** 2026-10-04

**Authors:** Alexander Caspary-Isar, Michael Jeanfavre, Olivia Kemp, Gretchen Leff, Mahin Rahman, Boglarka Huddleston

**Affiliations:** Stanford Health Care and Outpatient Orthopedic, Sports Medicine Rehabilitation Department, Redwood City, CA 94063, USA

**Keywords:** anterior cruciate ligament reconstruction, quadriceps strength, sex differences, muscle morphology, rate of torque development, rehabilitation

## Abstract

Background: Quadriceps strength deficit remains one of the most persistent impairments following anterior cruciate ligament reconstruction (ACLR) and is linked to delayed return to sport and increased reinjury risk. While females sustain ACL injuries at substantially greater rates than males, it remains unclear whether biol ogical sex also influences postoperative quadriceps recovery. The purpose of this systematic review was to determine whether sex-based differences exist in quadriceps strength, power, and morphology following ACLR in adults. Methods: This review was conducted in accordance with PRISMA guidelines and prospectively registered with PROSPERO. A comprehensive search of PubMed, CINAHL, SPORTDiscus, and Embase was performed to identify studies reporting sex-specific objective quadriceps outcomes regarding peak torque, peak torque-to-body weight ratio, rate of torque development, or muscle morphology in adults following primary unilateral ACLR. Risk of bias was assessed using the PEDro scale for randomized controlled trials and the Joanna Briggs Institute checklist for non-randomized studies. Results: Of the 1411 unique citations, seven studies met the inclusion criteria, comprising 552 participants (231 females, 321 males). Peak torque limb symmetry index (LSI) favored males in most studies, and peak torque normalized to body weight favored males in every comparison. When these absolute values are interpreted against sex-specific normative data, the male advantage appears inconsistent at 60°/s but consistent at 180°/s, suggesting a more nuanced pattern than raw strength values alone convey. Quadriceps power (rate of torque development) was assessed in only one study, which found a female disadvantage in isometric RTD limb symmetry. A second study assessed a related but mechanistically distinct outcome—the percentage decline in absolute torque after a fatiguing bout—and likewise found a greater decline in females. A single study assessing the morphology of the quadriceps found no sex difference in quad circumference within the ACLR participants. Conclusions: Sex-based quadriceps recovery deficits after ACLR are not uniform. A pattern consistent with a velocity- and fatigue-dependent female disadvantage was observed, but because only one study assessed true power/rate of torque development, testing velocity was confounded with graft type and other sample characteristics across studies, this pattern should be treated as a hypothesis rather than an established feature of sex differences after ACLR. Clinicians should reference strength outcomes against sex-specific normative data; whether incorporating fatigue- and velocity-sensitive testing into return-to-sport criteria improves outcomes has not been established by this body of evidence and warrants prospective study. Given the limited number of eligible studies, these findings should be considered hypothesis-generating.

## 1. Introduction

Anterior cruciate ligament (ACL) injury is one of the most common and complex injuries affecting individuals, with an estimated annual incidence of approximately 68.6 per 100,000 person-years in the general population and substantially greater rates among athletes participating in cutting and pivoting sports [1]. In the United States alone, more than 200,000 ACL injuries occur annually, with approximately 100,000–175,000 ACL reconstruction procedures performed each year [2,3]. Over the past two decades, evidence consistently demonstrates that female athletes experience a substantially greater risk of ACL injury than males participating in pivoting sports, with females sustaining ACL tears at rates of approximately two to eight times greater than males [4,5,6]. This disparity is particularly evident in sports such as soccer, basketball, handball, and volleyball, where females demonstrated disproportionately greater ACL injury rates despite similar exposure [7,8].

Given this elevated injury burden, Anterior Cruciate Ligament Reconstruction (ACLR) has become the most commonly performed procedure for restoring knee stability, improving daily function, as well as facilitating return to full performance following ACL injury. Although the advancements in surgical techniques, graft selection, rehabilitation strategies, as well as return-to-sport (RTS) battery testing have improved clinical outcomes, there continues to be persistent impairments in quadriceps strength and power [9,10]. Inadequate restoration of quadriceps strength has been associated with decreased functional performance, delayed return to sport, as well as increased risk of reinjury [11,12,13]. The quadriceps muscles play a fundamental role in stability, force generation, and absorption during movements requiring acceleration, change of direction, etc. Following ACL injury, quadriceps dysfunction develops through a multifactorial process that can include arthrogenic muscle inhibition (AMI), pain, joint effusion, and muscle atrophy [14]. These impairments can then lead to clinically meaningful deficits in quadriceps strength, rate of force development (RFD), and quadriceps size.

Quadriceps strength deficits following ACLR are most commonly quantified using isokinetic or isometric dynamometry with outcomes including peak torque, peak torque to body weight, limb symmetry index (LSI), and hamstring to quad ratio [15,16,17]. Although peak torque LSI remains one of the most frequently used RTS criteria, increased evidence has questioned its ability to accurately identify residual impairments because bilateral strength reduction may inflate symmetry values and mask persistent weakness [18]. Peak torque normalized to body weight provides an estimate of absolute quadriceps strength relative to an individual’s body size and can be interpreted against established normative reference values rather than relying solely on limb symmetry [19,20]. Beyond quadriceps strength, restoration of quadriceps power has emerged as an important predictor of athletic performance following ACLR. Rate of force development (RFD) reflects the ability of the neuromuscular system to generate force rapidly and is essential during explosive tasks such as jumping, cutting, pivoting, and sprinting [21]. Another important metric of postoperative recovery status is muscular morphology. Significant reductions in quadriceps cross-sectional area, muscle thickness, and muscle volume have been documented following ACLR and contribute to persistent weakness despite improvements in quadriceps activation [22]. Together, these strength, power and morphological measures capture distinct, but complementary dimensions of quadriceps recovery and evaluating them collectively provides a complete image of postoperative quadriceps function rather than a single outcome measure alone.

Beyond its established role in ACL injury risk, biological sex may also influence how patients recover following reconstruction. While the exact mechanisms underlying the greater incidence of ACL injury in females are multifactorial and remain incompletely understood, proposed contributors include anatomical differences, hormonal influences, neuromuscular control strategies, biomechanical movement patterns, joint laxity, and differences in baseline strength and power characteristics [23,24,25]. Recent research has further highlighted the potential role of menstrual cycle-related hormonal fluctuations, including changes in relaxin, estrogen, and progesterone concentrations, in influencing ligament properties and ACL injury susceptibility. Specifically, relaxin has been proposed to affect collagen remodeling and ligamentous laxity through interactions with ACL tissue, suggesting a potential biological mechanism contributing to sex-based differences in ACL injury risk [26]. Although these sex-specific risk factors have been extensively investigated with respect to ACL injury prevention, considerably less attention has been directed towards whether similar biological differences influence postoperative recovery following ACLR, particularly restoration of quadriceps strength, power, and morphology.

Several recent investigations that included pediatric populations in addition to adults have begun examining whether sex influences postoperative recovery [27,28,29,30]. Ageberg et al. [27] reported that females consistently demonstrated decreased patient-reported knee function and quality of life than males following ACLR, suggesting meaningful sex-related differences in recovery beyond restoration of mechanical knee stability. Kuenze et al. [29] demonstrated that females exhibited decreased quadriceps rate of torque development during the first postoperative year despite similar maximal strength, suggesting that recovery of neuromuscular function may differ from maximal force production. Similarly, Kim et al. [28] reported greater residual quadriceps weakness among females one year after surgery, supporting evidence of prolonged quadriceps inhibition in female patients. Hunnicutt et al. [30] found that female sex and 3-month extension ROM deficits were identified as significant predictors of 6-month quadriceps peak torque LSI deficits. Collectively, these investigations demonstrate that biological sex may influence recovery trajectories, however important gaps remain. Previous studies have largely focused on ACL injury risk, RTS outcomes in addition to including pediatric populations in the study, which could bias results due to not reaching full development. Clinicians currently lack a comprehensible synthesis of available evidence regarding quadriceps outcomes to determine whether rehabilitation expectations, strength benchmarks, and RTS criteria should differ between males and females. The purpose of this systematic review was to determine whether sex-based differences exist in quadriceps strength, power, and morphology following ACL reconstruction, specifically in adults.

## 2. Materials and Methods

This systematic review was conducted in accordance with the Preferred Reporting Items for Systematic Reviews and Meta-Analyses (PRISMA) guidelines [31,32,33]. The PRISMA checklist was used to guide the reporting of study identification, screening, eligibility, and inclusion, as well as data extraction and synthesis. A priori protocol was developed in accordance with PROSPERO recommendations and prospectively registered with the International Prospective Register of Systematic Reviews (PROSPERO; Registration No. CRD420261338598).

### 2.1. Study Eligibility Criteria

The framework for this study was developed using the Population, Exposure, Comparison, and Outcome (PECO) format [34,35]. The inclusion and exclusion criteria based on the PECO question are presented in Table 1. Eligible studies included adult populations undergoing anterior cruciate ligament reconstruction. Studies were required to provide sex-specific comparisons and report at least one of the following primary outcomes: quadriceps peak torque, quadriceps peak torque normalized to body weight, rate of force development, or quadriceps girth.

### 2.2. Study Identification and Search Strategy

A comprehensive literature search was conducted in January–March 2026 across the PubMed, CINAHL, SPORTDiscus, and Embase databases to identify studies relevant to the review question. The search strategy was developed in collaboration with a health sciences librarian at Stanford University Medical Library to optimize database-specific search syntax, ensure appropriate use of Boolean operators, and maximize the sensitivity and specificity of the search process. Search terms were derived from the key concepts of the review and included variations of “anterior cruciate ligament reconstruction,” “quadriceps,” and “sex differences.” The PubMed search string, as executed in Covidence, is presented in Figure 1. The complete PubMed search strategy, as well as database-specific search strategies for CINAHL, SPORTDiscus, and Embase, are provided in Appendix A. To further enhance the comprehensiveness of the search, a manual review of reference lists from eligible studies was performed. Additional relevant articles identified through citation tracking and the study screening process were also considered for inclusion. Screening was restricted to studies published in English or with an accessible translation because the review team could not reliably appraise methodological quality, extract numeric outcome data, or resolve ambiguous terminology in languages the reviewers do not read.

**Figure 1:**
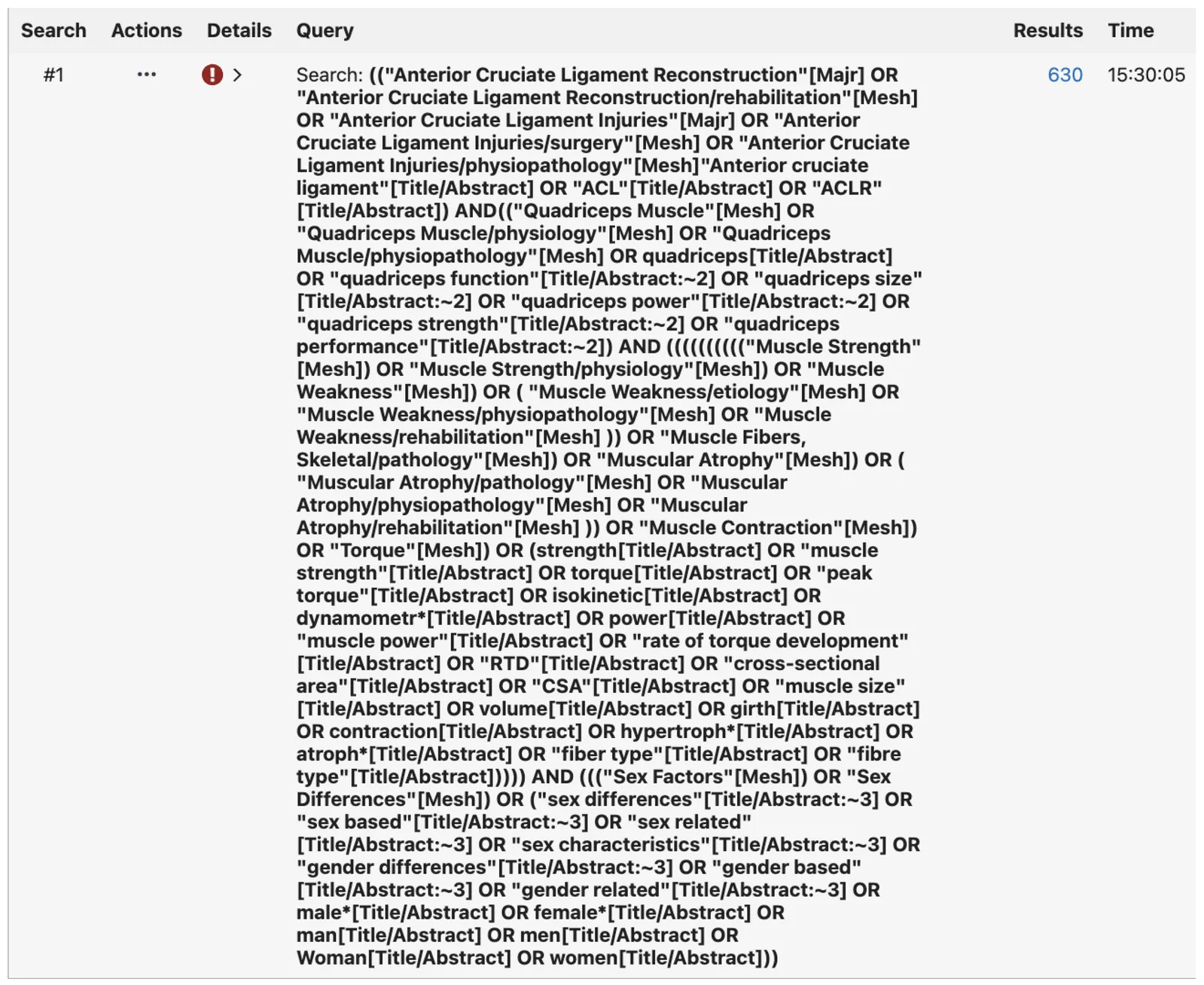
Search strategy (PubMed search string shown as executed in Covidence).

### 2.3. Study Selection

Records retrieved from all databases were imported into a single library and underwent deduplication before screening. Study selection was completed independently by two reviewers (AC and OK) using Covidence systematic review software (Veritas Health Innovation, Melbourne, Australia). Titles, abstracts, and full-text articles were evaluated against the predetermined eligibility criteria. Any disagreements regarding study eligibility were resolved through discussion and consensus between the two reviewers (AC and OK). Following review of the available evidence and application of the inclusion and exclusion criteria, all discrepancies were successfully reconciled without the involvement of an additional reviewer. A notable proportion of full-text articles were excluded as study participants were younger than 18 years of age. Given that the objective of this review was to examine sex-based differences in quadriceps outcomes among adults following anterior cruciate ligament reconstruction, studies involving pediatric and adolescent populations were not considered eligible for inclusion.

### 2.4. Data Extraction

The variables extracted from the full-text articles were defined based on the study’s inclusion and exclusion criteria to ensure consistency across studies and to directly address the PECO question. Data extraction was organized into three primary areas: patient demographics, methodological characteristics, and outcome results. For the patient demographic domain, extracted variables included sex distribution of participants, mean age with corresponding ranges or standard deviations, graft type used in ACL reconstruction, and time since surgery at the point of outcome assessment. In addition, relevant baseline and clinical characteristics were recorded, including activity level, sport participation status, and any other reported outcomes, even if not directly specific to quadriceps performance, to provide appropriate contextual understanding of study populations and potential sources of heterogeneity. For the methodological data, detailed information was extracted regarding timing of postoperative assessment, the assessment tools and protocols used to evaluate quadriceps function were also documented, including isokinetic dynamometry parameters, isometric testing protocols, and any imaging-based or anthropometric methods used to quantify quadriceps morphology. When available, additional details such as procedures, testing reliability measures, and standardization protocols were extracted to support appraisal of methodological quality and comparability across studies. For the results data, extraction focused on quadriceps-specific measures aligned with the objectives of the review. This structured and standardized extraction process ensured that all relevant quantitative and qualitative data were consistently captured across studies, enabling meaningful synthesis of sex-based differences in quadriceps outcomes following ACL reconstruction.

### 2.5. Summary Measures and Synthesis of Results

Outcomes were grouped into the three predefined domains of quadriceps strength, quadriceps power, and quadriceps morphology. Within each domain, the studies were compared according to the objective outcome measure reported. When available, the descriptive statistics and measures of statistical significance were extracted to summarize the directions and consistency among the studies. The synthesis emphasizes patterns of agreement and disagreement between studies while accounting for study design, methodology, and postoperative follow-up duration. The results were then organized into multiple graphical displays to summarize the primary outcomes (AC).

### 2.6. Normative-Referenced Secondary Analysis

Peak torque to body weight (Nm/kg) is known to differ between sexes, independent of injury. Due to this important notion, the peak torque to body weight values were re-expressed as a percentage of published sex-specific normative values to distinguish recovery deficits from pre-existing strength differences. Two of three candidate published reference datasets were ultimately used to re-express peak torque to body weight as a percentage of normative capacity, each matched to an included study’s testing velocity and protocol. Isokinetic values at 60°/s and 180°/s were referenced against Van Melick et al.’s [19] scoping review, which pooled quadriceps and hamstring strength reference values from 42 primary studies of competitive pivoting-sport athletes (soccer, basketball, handball, and other pivoting sports). This reference band is itself a synthesis across many original samples rather than a single cohort with one defined age range or competition level, it carries its own internal heterogeneity in addition to any mismatch with this review’s ACLR samples. Isometric torque at approximately 80–90° knee flexion was referenced against Barrué-Belou et al.’s [36] single cohort of 74 trained, uninjured adults (35 men, 39 women; mean age 24.1 ± 5.7 years, range 18–41) who competed in ACL-injury-risk sports. A third candidate dataset, Šarabon et al.’s [37] meta-analysis of isometric knee extension and flexion strength, was considered but not used for any comparison in this review: its pooled values are drawn predominantly from general, non-athletic adult and older-adult populations rather than sport-specific cohorts, and are reported only for extended, mid-range, and flexed knee-angle positions rather than the isokinetic velocities or the specific isometric angle used by the included ACLR studies, so no included outcome could be matched to it on the same basis used for the other two datasets. Because the ACLR cohorts were post-surgical patients of variable age and activity level, while the normative comparators were themselves either a pooled synthesis of competitive athletes (Van Melick) or a single young, trained, uninjured cohort (Barrué-Belou), a difference between sexes in percentage of normative capacity could reflect a genuine sex-based recovery difference, an underlying mismatch in age, sport participation, or training status between the ACLR sample and its matched normative comparator, or both. This analysis should therefore be interpreted as exploratory. Confidence intervals around these derived percentages could not be calculated because the normative datasets did not report the variance needed to propagate uncertainty.

### 2.7. Risk-of-Bias Assessment

Consistent with the Cochrane Handbook [38], the risk of bias and quality appraisal of the included RCTs and non-RCTs were assessed. The risk-of-bias assessment (RoB) of included studies was performed using the PEDro (Physiotherapy Evidence Database) scale for randomized controlled trials and the Joanna Briggs Institute (JBI) critical appraisal for non-randomized control trials. The JBI quality appraisal tool was used for all non-randomized controlled trials, with the majority being cohort studies. The revised JBI critical appraisal checklist was developed to incorporate advancements in risk-of-bias methodology, enhancing its applicability and relevance for systematic reviews. The updated tool enables reviewers to evaluate both methodological quality and potential sources of bias in studies, supporting a more comprehensive assessment of study quality during the systematic review process [39].

The method of scoring was eleven questions, with ten being scored, featuring answers of yes, no, or unclear for cohort and case series. For this review, unclear will be treated as no. The scale utilized for the ten questions JBI checklist will be 8–10 as “low risk”, 5–7 as “moderate risk,” 0–4 as “high risk”. For studies assessed using the 8-item JBI critical appraisal checklist, scores of 7–8 were classified as “low risk”, scores of 5–6 as “moderate risk”, and scores of 0–4 as “high risk”. For studies assessed using the 9-item JBI critical appraisal checklist, scores of 8–9 were classified as “low risk”, scores of 5–7 as “moderate risk”, and scores of 0–4 as “high risk”. These thresholds were established by this review’s authors to facilitate interpretation of study quality, as the JBI does not provide standardized scoring cutoffs. The PEDro scale is an 11-item instrument designed to evaluate the methodological quality and internal validity of clinical trials, with 10 items contributing to the final score (range 0–10). Greater scores indicate greater quality of methods. The scale assesses domains including random allocation, concealed allocation, blinding, adequacy of follow-up, intention-to-treat analysis, and statistical reporting [40]. Studies scoring 9–10 were classified as “excellent quality”, 6–8 as “good quality”, 4–5 as “fair quality”, and scores below 4 as “poor quality”. RoB assessments for RCTs and non-RCTs were performed by two independent research members (Author 1: A.C., Author 2: O.K.). No discrepancies were identified between the two reviewers; a third reviewer was not needed.

### 2.8. Level of Evidence and Certainty

The methodological quality of the included studies was evaluated using the Oxford Centre for Evidence-Based Medicine (OCEBM) Levels of Evidence framework (Table 2). The Grading of Recommendations Assessment, Development and Evaluation (GRADE) framework (Table 3, Appendix B) is presented to describe the general approach used to reason about certainty of evidence in systematic reviews; outcome-specific GRADE ratings were not generated for this review’s individual outcomes given the small number and marked heterogeneity of the included studies, and the certainty of the body of evidence is instead characterized narratively in Section 4.3. These appraisal methods were selected to provide a standardized and transparent assessment of both individual study quality and the overall strength of the evidence synthesized in this review. The OCEBM framework was used to classify studies according to their methodological design and level of evidence. Because the risk-of-bias assessment (Section 2.7) and the OCEBM study-design classification already characterize the design-related sources of uncertainty in this evidence base, and because outcome-specific GRADE ratings were not generated (see above), the additional interpretive value of the OCEBM classification beyond the risk-of-bias tables is limited. It is retained here for transparency and comparability with prior reviews. The certainty of evidence for the review’s outcomes is instead discussed narratively—considering study design, risk of bias, consistency, precision, and directness—in Section 4.3 [41,42].

## 3. Results

### 3.1. Study Selection and Characteristics

Following the removal of duplicate records using Covidence, the initial database search yielded 1411 unique articles for screening. Titles and abstracts of all 1411 studies were reviewed for relevance based on the predetermined inclusion and exclusion criteria. Studies that met the eligibility criteria, or for which eligibility could not be determined from the title and abstract alone, underwent full-text review. Following the full-text screening process, seven studies met all inclusion criteria and were included in the final qualitative synthesis. The seven included studies represented a range of study designs, comprising one randomized controlled trial (RCT), three cohort studies, one case series, one cross-sectional study, and one quasi-experimental study. The study selection process, including the identification, screening, eligibility assessment, and final inclusion of studies, is summarized in the PRISMA flow diagram presented in Figure 2.

### 3.2. Participant Demographics

The total number of participants in this study is 552 participants with 231 females and 321 males, ranging from 18 to 55 years old (Table 4). The majority of studies contained sample sizes ranging from 26 to 140 participants per study, with most participants being categorized as young-middle-aged adults. The reported mean ages were 29.2 for females and 28.2 for males, demonstrating comparable age distribution between sexes. The included studies represented a diverse range of research methodologies, consisting of one randomized controlled trial, three cohort studies, one retrospective case series, one cross-sectional study, and one quasi-experimental study with a pretest–posttest design. The participants included were predominantly recreationally active individuals or athletes who participated in cutting, pivoting, and jumping sports. Follow-up periods varied, ranging from 3 months to 5 years post ACL reconstruction, reflecting both the early and later stages of the postoperative recovery. Graft type was reported in six of the seven included studies and demonstrated considerable variability across the study populations. Hamstring-based autografts were the most frequently utilized graft choice, accounting for at least 242 reconstructions (43.8%). Bone–patellar tendon–bone (BPTB) autografts were performed in at least 36 participants (6.5%), while quadriceps tendon autografts were utilized in 48 participants (8.7%). Allografts were reported in seven participants (1.2%), including both patellar tendon and unspecified allografts. Additionally, 65 participants (11.8%) underwent reconstruction using a combined central one-third patellar tendon and quadriceps tendon autograft technique, and graft type was not reported for the remaining participants. Overall, hamstring-based autografts represented the predominant reconstruction method among the included studies, with substantially fewer participants receiving quadriceps tendon autografts, BPTB autografts, allografts, or combined graft techniques.

**Table 4:** Study demographics.

First Author (Year)	Study Design	Activity Level	Setting/ Population	Sample (N | sex, n | Age, y, Mean ± SD)	Graft Type (n)	Comparison Groups
Ebert et al. [42]	Randomized controlled trial	Pivoting-sport participation	Adult ACLR (Australia); orthopedic center	N = 97 F 50 | age 29.8 ± 7.8 M 47 | age 28.0 ± 8.2	Hamstring tendon (HT) autograft: 49 Quadriceps tendon (QT) autograft: 48	Female HT (n = 25)/Female QT (n = 25) Male HT (n = 24)/Male QT (n = 23)
Kuenze et al. [43]	Quasi-experimental (pre/post-exercise, cross-sectional)	Recreationally active (≥3 sessions/wk, ≥30 min/session)	University community; laboratory setting	N = 26 F 13 | age 24.2 ± 5.6 M 13 | age 24.1 ± 4.4	Patellar tendon (PT) autograft: 8 Hamstring tendon (HT) autograft: 15 Allograft: 3	Female pre-exercise/Female post-exercise Male pre-exercise/Male post-exercise
Otzel et al. [44]	Cross-sectional (within- and between-subject)	Recreationally/physically active university population	University-based cohort (USA); ~3 y post-op	N = 24 ACLR (+23 healthy controls) F 13 (ACLR) | age 20.2 ± 1.1 M 11 (ACLR) | age 21.3 ± 2.5	PT autograft: 18 HT autograft: 2 PT allograft: 4	ACLR Female/ACLR Male Control Female/Control Male
San Jose et al. [45]	Prospective observational cohort	Recreationally active, pivoting-sport participation	Sports-medicine clinic (USA); two post-op windows	N = 89 F 38 | age 23 (range 20–29) M 51 | age 21 (range 18–25)	Semitendinosus autograft: 47 Semitendinosus + gracilis autograft: 42	Female/Male at 3–6 mo post-op Female/Male at 7–12 mo post-op
Zore et al. [46]	Retrospective cohort	Pivoting-sport athletes	Pandemic-era single-center cohort; strata by year of surgery	N = 126 F 56 | age 36.5 ± 12.6 M 70 | age 33.8 ± 11.1	Hamstring autograft: 126	Pre-pandemic control/2020/2021/2022 (within-sex, across four cohorts)
Yasuda et al. [47]	Prospective comparative observational cohort	Mixed; sport or daily activity limited pre-op	Orthopedic follow-up cohort (Japan)	N = 65 followed; 50 analyzed for strength F 21 | M 29 Age 23 y (range 18–39, whole cohort)	Combined central-third patellar tendon + quadriceps tendon autograft	Female/Male at pre-op, 3, 6, 9, 12 mo, and 4.5 y final follow-up (range 3–7)
Rips et al. [48]	Retrospective case series	Recreationally/physically active adult ACLR cohort	Orthopedic follow-up cohort; stratified by TSK	N = 140 F 40 | age 33.0 ± 7.4 M 100 | age 31.3 ± 10.1	Male BTB autograft: 26 Male HT autograft: 74 Female BTB autograft: 2 Female HT autograft: 38	Female/Male, stratified by TSK > 37 vs. TSK ≤ 37
Aggregate (k = 7)	1 Level II, 5 Level III, 1 Level IV (OCEBM)	Predominantly pivoting-sport/recreationally active	USA, Australia, Japan, mixed clinical + laboratory	N = 552 F 231 | weighted mean age 29.2 y M 321 | weighted mean age 28.2 y	HT: 253; PT: 26; QT: 48 BTB: 28; Allograft: 3; Combined: 50 (incl. 126 HT from Zore)	Sex is the review-level exposure across all studies

Abbreviations: ACLR, anterior cruciate ligament reconstruction; BTB, bone–patellar tendon–bone; F, female; HT, hamstring tendon; M, male; PT, patellar tendon; QT, quadriceps tendon; RCT, randomized controlled trial; SD, standard deviation; TSK, Tampa Scale of Kinesiophobia.

### 3.3. Study Methodology

The seven studies that were included in this systematic review demonstrated methodological heterogeneity with respect to study design, timing of postoperative assessment, and the specific quadriceps outcome measures (Table 5). Assessment timelines varied across the studies, reflecting a spectrum of postoperative recovery following ACL reconstruction. Participants were evaluated as early as three months following surgery and up to five years. Although outcome measures varied among the studies, the isokinetic dynamometer represented the primary method of strength assessment, with six of the seven studies utilizing isokinetic dynamometers to assess quadriceps performance. Peak torque was the most frequently evaluated measure during concentric extension at angular velocities of 60°/sec, 90°/sec, and 180°/sec. The studies also reported peak torque limb symmetry index (LSI) as well as peak torque to body weight. Rate of torque development was also assessed via isokinetic and isometric measurements. Only one study included quadriceps morphology that ultimately used standardized quadriceps size circumference, and did not utilize any advanced imaging modalities. The reliability of the assessment methods was consistent across the included studies, with isometric/isokinetic assessments for Biodex, Humac Norm, KinCom, Cybex, and Isosport demonstrating excellent reliability of ICC greater than 0.85 supporting the validity of objective quadriceps strength measurements across studies, despite the methodological heterogeneity between studies [45,46,47,48]. Overall, the included studies demonstrate consistent use of objective and reliable methods to assess postoperative quadriceps outcome measures. The methodological variability will be considered when synthesizing the results regarding the outcomes in question.

**Table 5:** Study methodology.

First Author (Year)	Study Design	Outcome Construct and Metric Class	Assessment Timeline (Post-op)	Instrumentation and Test Protocol	Reliability
Ebert et al. [42]	Randomized controlled trial	Quadriceps peak torque (absolute-capacity) Knee extensor LSI (recovery-referenced)	12 mo 24 mo	Isosport International/Gepps Cross isokinetic dynamometer–90°/s (concentric knee extension/flexion).	Isosport ICC: 0.85–0.98
Kuenze et al. [43]	Quasi-experimental (pre/post-exercise)	MVIC torque (absolute-capacity) Superimposed-burst torque (absolute-capacity)	F: 45.8 ± 42.7 mo M: 43.5 ± 37.0 mo (pre and post standardized exercise)	Biodex multi-modal dynamometer; superimposed burst compared with the 250-ms pre-stimulation average.	Biodex ICC: 0.86–0.98
Otzel et al. [44]	Cross-sectional (within- and between-subject)	Peak torque/body mass (absolute-capacity) Involved-limb deficit (recovery-referenced) Thigh circumference (morphology)	F: 2.5 ± 1.5 y M: 3.3 ± 1.8 y	KinCom AP125 isokinetic dynamometer–180°/s then 60°/s, side order randomized. Thigh circumference measured with flexible tape at the largest visible mid-belly, referenced to the superior patellar border.	Thigh circumference ICC: 0.85–0.99 KinCom ICC: 0.90–0.99
San Jose et al. [45]	Prospective observational cohort	Concentric MVC LSI (recovery-referenced) Isometric MVC LSI (recovery-referenced) Isometric RTD LSI (recovery-referenced)	3–6 mo 7–12 mo	Humac isokinetic dynamometer; 5-min bike warm-up. Isometric: 3-s max push, 3 reps/muscle. Concentric: 5 max reps with verbal + visual feedback after.	Humac ICC: 0.81–.97
Zore et al. [46]	Retrospective cohort	Peak torque (absolute-capacity) Peak torque/body weight (absolute-capacity) Quadriceps asymmetry index (recovery-referenced—inverse of LSI)	F: 22.8 ± 7.0 wk M: 22.5 ± 4.7 wk	Humac Norm isokinetic dynamometer–60°/s. Q-AI = (uninjured − injured)/uninjured × 100–inverse of LSI, higher Q-AI = worse.	Humac ICC: 0.81–.97
Yasuda et al. [47]	Prospective comparative observational cohort	Isometric peak torque LSI (recovery-referenced)	Pre-op; 3, 6, 9, 12 mo; final 4.5 y (range 3–7)	Cybex II dynamometer–isometric at 70° flexion. LSI expressed as ratio of injured to uninjured limb (× 100).	Cybex II ICC: 0.85–0.98
Rips et al. [48]	Retrospective case series	Peak torque LSI (recovery-referenced) Peak torque/body weight (absolute-capacity)	F: 5.4 ± 1.3 y M: 5.5 ± 1.2 y	Humac Norm isokinetic dynamometer. 60°/s: 3 trials, 3 test reps; 180°/s: 5 trials, 15 test reps; inter-trial rest 2 min, inter-speed 1 min, inter-limb 2 min.	Humac ICC: 0.81–0.97

Abbreviations: LSI, limb symmetry index; MVC, maximal voluntary contraction; MVIC, maximal voluntary isometric contraction; Q-AI, quadriceps asymmetry index; RTD, rate of torque development; SIB, superimposed burst; ICC, intraclass correlation coefficient.

### 3.4. Risk-of-Bias Assessment

The risk-of-bias assessment results are summarized in Table 6A–E. The PEDro scale utilized for randomized controlled trials is displayed in Table 6A. The Joanna Briggs Institute (JBI) critical appraisal was utilized for all observational studies and is displayed in Table 6B–E.

The randomized controlled trial by Ebert et al. [49] received a score of five due to a lack of participant and assessor blinding as well as the absence of concealed allocation. The lack of blinding in rehabilitation and physical therapy literature is well documented and the risk-of-bias assessment results in this review further corroborate this limitation [50].

Among the cohort studies, the JBI appraisal scores ranged from 7–10, with two studies classified as low risk (San Jose [51]; Zore [52]) and one study classified as moderate risk (Yasuda [53]). The primary areas that deducted points were related to identification and control of confounding variables, as well as completeness of follow-up reporting. The cross-sectional study by Otzel [54] received a JBI score of 7 out of 8, corresponding to low risk of bias, with the primary limitation being the lack of identification and management of confounding variables. The quasi-experimental study by Kuenze [55] received a JBI score of 8 out of 9, indicating low risk, with the primary limitation being potential bias related to participant group comparisons. The case series by Rips [56] received a JBI score of 8 out of 10, also classified as low risk, with the primary limitations related to participant selection and reporting of demographic characteristics.

### 3.5. Summary Measures and Synthesis of Results

#### 3.5.1. Quadriceps Strength: Peak Torque Limb Symmetry Index (LSI)

Across the four studies reporting peak torque LSI, males demonstrated greater limb symmetry than females at nearly every significant timepoint with the sole exception of Otzel’s 180°/s within-limb deficit comparison, which favored females. Ebert et al. [49] reported LSI (90°/s) of 78.6–89.1% (female) versus 86.0–94.7% (male) across 12- and 24-month follow-up, favoring males at both timepoints (*p* ≤ 0.010). San Jose [51] reported concentric LSI of 75–76% versus 81–83% at 60°/s and 180°/s (*p* ≤ 0.001, favoring males), while isometric MVC LSI (86 vs. 92%) was non-significant. Yasuda et al. [53] reported isometric LSI (70°) rising from 78% (female) vs. 79% (male) pre-operatively (non-significant) to 70% vs. 85% at final follow-up (mean 4.5 years), favoring males at every postoperative timepoint (*p* < 0.001). Otzel et al. [54], in a small cohort (13 females, 11 males), reported an involved-limb deficit favoring males at 60°/s (9% vs. 6%) but favoring females at 180°/s (7% vs. 8%; both *p* < 0.05) as displayed in Figure 3 below.

#### 3.5.2. Quadriceps Strength–Peak Torque Normalized to Body Weight

Raw peak torque relative to body mass favored males across all four studies reporting absolute strength outcomes. Among studies reporting isokinetic torque, this pattern was observed at both 60°/s (Figure 4) and 180°/s (Figure 5). Kuenze et al. [55] found no baseline sex differences in absolute torque for either MVIC (2.56 vs. 2.63 Nm/kg) or SIB (3.39 vs. 3.49 Nm/kg); however, females demonstrated significantly greater post-exercise declines in MVIC (*p* = 0.02) and SIB (*p* = 0.04). Otzel et al. [54] reported torque values ranging from 1.18 to 1.86 Nm/kg in females and from 1.86 to 2.55 Nm/kg in males across testing velocities and limbs (all *p* < 0.001). Zore et al. [52] reported involved-limb torque at 60°/s ranging from 1.38 to 1.82 Nm/kg in females and from 2.00 to 2.20 Nm/kg in males across the pre-pandemic control and 2020–2022 surgical-year cohorts (Figure 4). Rips et al. [56] reported torque values ranging from 1.14 to 1.88 Nm/kg in females and from 1.45 to 2.21 Nm/kg in males across the Tampa Scale of Kinesiophobia (TSK) group. Males demonstrated greater torque in three of four comparisons (*p* ≤ 0.006), whereas the 60°/s comparison within the TSK > 37 group was not significant. The study also reported a substantial sex-by-graft imbalance, with a greater proportion of males receiving BTB grafts.

#### 3.5.3. Quadriceps Power

One study assessed quadriceps power as defined by this review’s PECO criteria. San Jose et al. [51] reported isometric rate of torque development (RTD) LSI of 81 ± 32% for females versus 89 ± 25% for males (*p* = 0.017), a statistically significant relationship favoring males in RTD recovery following ACLR, displayed in Figure 6. A second study, Kuenze et al. [55], did not measure power or RTD; it assessed the percentage decline in absolute isometric torque (MVIC and superimposed-burst) following a standardized fatiguing exercise bout. Kuenze et al. [55] found equivalent pre-exercise superimposed-burst torque between sexes (3.39 vs. 3.49 Nm/kg), but torque declined significantly more in females than males after fatigue (−10.32% vs. −1.45%, *p* = 0.02). This fatigue-induced torque decline is a related but distinct construct from power/RTD and should not be pooled with it as though both measured the same domain; together these two studies contribute one power/RTD data point and one fatigability data point to this review’s evidence base, both shown in Figure 6.

#### 3.5.4. Quadriceps Morphology

A singular study by Otzel et al. [54] reported morphology data in which the quadriceps circumference did not differ by sex within the ACLR cohort. It did differ, favoring males in healthy uninjured controls. Otzel et al. [54] reported involved-limb circumference of 51 cm (female) versus 53 cm (male), or 96.4% and 98.5% of normative values for active individuals [57], respectively (*p* = 0.442, no difference), and uninvolved-limb circumference of 52 cm versus 53 cm, or 98.3% and 98.5% of normative values. Circumference is an indirect surrogate for muscle size that cannot distinguish contractile tissue from subcutaneous or connective tissue and provides no information about architectural properties such as pennation angle, fascicle length, or muscle thickness, which vary by sex and contribute independently to force-generating capacity [58]. Because circumference was the only morphological measure available across the included studies, this review’s morphology findings should be interpreted as a limited window into a construct more completely characterized using imaging-based architectural assessment. Sex-based quadriceps outcomes from all seven included studies are summarized by metric class in Table 7.

**Table 7:** Sex-based quadriceps outcomes grouped by metric class.

Study	Metric Class	Outcome/Condition	Time Point	Female (Mean ± SD)	Male (Mean ± SD)	Statistical Result	Direction
**BLOCK 1—QUADRICEPS STRENGTH: LIMB SYMMETRY INDEX**							
Ebert et al. [42]	LSI, 90°/s	Knee extensor peak-torque LSI, all grafts pooled	12 mo	78.6 ± 16.6%	86.0 ± 13.1%	Sex effect *p* = 0.010; time effect *p* < 0.0001	Favors males
Ebert et al. [42]	LSI, 90°/s	Knee extensor peak-torque LSI, all grafts pooled	24 mo	89.1 ± 11.8%	94.7 ± 7.8%	Group effect *p* = 0.001; time × group *p* = 0.02	Favors males
Ebert et al. [42]	LSI, 90°/s	Knee extensor LSI—HT autograft	12 mo	82.9 ± 14.1%	88.2 ± 11.7%	See pooled model above	Favors males
Ebert et al. [42]	LSI, 90°/s	Knee extensor LSI—QT autograft	12 mo	75.2 ± 17.9%	82.6 ± 13.1%	See pooled model above	Favors males
Ebert et al. [42]	LSI, 90°/s	Knee extensor LSI—HT autograft	24 mo	91.0 ± 9.9%	94.9 ± 5.3%	See pooled model above	Favors males
Ebert et al. [42]	LSI, 90°/s	Knee extensor LSI—QT autograft	24 mo	87.3 ± 12.2%	91.0 ± 9.5%	See pooled model above	Favors males
San Jose et al. [45]	LSI, 60°/s	Concentric quadriceps MVC LSI	3–6 and 7–12 mo (combined)	75 ± 17%	81 ± 15%	*p* = 0.001	Favors males
San Jose et al. [45]	LSI, 180°/s	Concentric quadriceps MVC LSI	3–6 and 7–12 mo (combined)	76 ± 14%	83 ± 13%	*p* < 0.001	Favors males
San Jose et al. [45]	LSI, isometric	Isometric quadriceps MVC LSI	3–6 and 7–12 mo (combined)	86 ± 20%	92 ± 19%	NS (no formal *p* reported)	Non-significant
San Jose et al. [45]	LSI, isometric RTD	Isometric quadriceps RTD LSI	3–6 and 7–12 mo (combined)	81 ± 32%	89 ± 25%	*p* = 0.017	Favors males
Yasuda et al. [47]	LSI, isometric 70°	Isometric quadriceps LSI	Pre-op	78 ± 8%	79 ± 9%	NS	No difference
Yasuda et al. [47]	LSI, isometric 70°	Isometric quadriceps LSI	3 mo	33 ± 5%	51 ± 10%	*p* < 0.001	Favors males
Yasuda et al. [47]	LSI, isometric 70°	Isometric quadriceps LSI	12 mo	66 ± 10%	78 ± 11%	*p* < 0.001	Favors males
Yasuda et al. [47]	LSI, isometric 70°	Isometric quadriceps LSI	4.5 y (range 3–7)	70 ± 9%	85 ± 13%	*p* < 0.001	Favors males
Otzel et al. [44]	Involved-limb deficit, 60°/s	Peak-torque within-limb deficit	~3 y	9% deficit	6% deficit	*p* < 0.05	Favors males
Otzel et al. [44]	Involved-limb deficit, 180°/s	Peak-torque within-limb deficit	~3 y	7% deficit	8% deficit	*p* < 0.05	Favors females
**BLOCK 2—QUADRICEPS PEAK TORQUE NORMALIZED TO BODY MASS**							
Kuenze et al. [43]	Nm/kg, MVIC	Quadriceps MVIC torque—pre-exercise	~44 mo	2.56 ± 0.70 Nm/kg	2.63 ± 0.68 Nm/kg	NS between sexes at baseline	No difference
Kuenze et al. [43]	Nm/kg, MVIC	Quadriceps MVIC torque—post-exercise	~44 mo	2.28 ± 0.65 Nm/kg	2.62 ± 0.55 Nm/kg	% reduction: F −10.32%, M −1.45%; *p* = 0.02	Favors males
Kuenze et al. [43]	Nm/kg, SIB	Superimposed-burst torque—pre-exercise	~44 mo	3.39 ± 0.40 Nm/kg	3.49 ± 0.82 Nm/kg	NS (not formally reported)	No difference
Kuenze et al. [43]	Nm/kg, SIB	Superimposed-burst torque—post-exercise	~44 mo	3.33 ± 0.53 Nm/kg	3.60 ± 0.82 Nm/kg	Group × time interaction *p* = 0.04	Favors males
Otzel et al. [44]	Nm/kg, 60°/s	Peak torque/body mass—involved limb	~3 y	1.77 Nm/kg	2.45 Nm/kg	*p* < 0.001	Favors males
Otzel et al. [44]	Nm/kg, 60°/s	Peak torque/body mass—uninvolved limb	~3 y	1.86 Nm/kg	2.55 Nm/kg	*p* < 0.001	Favors males
Otzel et al. [44]	Nm/kg, 180°/s	Peak torque/body mass—involved limb	~3 y	1.18 Nm/kg	1.86 Nm/kg	*p* < 0.001	Favors males
Otzel et al. [44]	Nm/kg, 180°/s	Peak torque/body mass—uninvolved limb	~3 y	1.28 Nm/kg	2.06 Nm/kg	*p* < 0.001	Favors males
Zore et al. [46]	Nm/kg, 60°/s	Peak torque/body weight—control cohort, involved	~5 mo	1.79 ± 0.42 Nm/kg	2.00 ± 0.70 Nm/kg	F involved *p* = 0.049 across yrs; M involved *p* = 0.809	Favors males
Zore et al. [46]	Nm/kg, 60°/s	Peak torque/body weight—2020, involved	~5 mo	1.48 ± 0.44 Nm/kg	2.20 ± 0.50 Nm/kg	See above	Favors males
Zore et al. [46]	Nm/kg, 60°/s	Peak torque/body weight—2021, involved	~5 mo	1.38 ± 0.52 Nm/kg	2.10 ± 0.50 Nm/kg	See above	Favors males
Zore et al. [46]	Nm/kg, 60°/s	Peak torque/body weight—2022, involved	~5 mo	1.82 ± 0.57 Nm/kg	2.00 ± 0.70 Nm/kg	See above	Favors males
Rips et al. [48]	Nm/kg, 180°/s	Peak torque/body weight, TSK > 37	F 5.4 y; M 5.5 y	1.18 ± 0.30 Nm/kg	1.45 ± 0.33 Nm/kg	*p* = 0.006	Favors males
Rips et al. [48]	Nm/kg, 180°/s	Peak torque/body weight, TSK ≤ 37	F 5.4 y; M 5.5 y	1.14 ± 0.20 Nm/kg	1.57 ± 0.33 Nm/kg	*p* < 0.001	Favors males
Rips et al. [48]	Nm/kg, 60°/s	Peak torque/body weight, TSK > 37	F 5.4 y; M 5.5 y	1.88 ± 0.41 Nm/kg	2.14 ± 0.47 Nm/kg	NS	Favors males
Rips et al. [48]	Nm/kg, 60°/s	Peak torque/body weight, TSK ≤ 37	F 5.4 y; M 5.5 y	1.84 ± 0.34 Nm/kg	2.21 ± 0.51 Nm/kg	*p* = 0.001	Favors males
**BLOCK 3—QUADRICEPS MORPHOLOGY**							
Otzel et al. [44]	Morphology	Thigh circumference—involved limb (ACLR)	~3 y	51 ± 0.5 cm	53 ± 0.5 cm	*p* = 0.442	No difference
Otzel et al. [44]	Morphology	Thigh circumference—uninvolved limb (ACLR)	~3 y	52 ± 0.4 cm	53 ± 0.5 cm	Not formally tested vs. sex	No difference
Otzel et al. [44]	Morphology	Thigh circumference—healthy controls (avg L/R)	n/a	52 ± 0.6 cm	57 ± 0.4 cm	*p* < 0.05	Favors males (uninjured knees)

Abbreviations: F, female; ICC, intraclass correlation coefficient; LSI, limb symmetry index; M, male; MVC, maximal voluntary contraction; MVIC, maximal voluntary isometric contraction; NS, non-significant; Nm/kg, newton-meters per kilogram body mass; Q-AI, quadriceps asymmetry index; RTD, rate of torque development; SD, standard deviation; SIB, superimposed burst; TSK, Tampa Scale of Kinesiophobia.

## 4. Discussion

### 4.1. Summary of Main Results

Quadriceps muscle dysfunction is one of the most persistent and clinically consequential impairments following ACLR. This is clinically important due to return-to-sport (RTS) criteria and rehabilitation dosing, which are frequently applied uniformly across sexes. This review systematically identified, appraised, and synthesized the available adult-only evidence addressing this question. The purpose was to determine whether sex-based differences exist in objective measures of quadriceps strength, power, and morphology after unilateral ACLR, and where differences were identified to then characterize their direction, consistency, and clinical implications.

Seven studies met the inclusion criteria, collectively enrolling 552 participants, of which 231 were females, and 321 were males. No single hypothesis of uniform female disadvantage, uniform equivalence, or male disadvantage can be accepted or rejected outright. Symmetry-referenced outcomes for peak torque LSI were inconsistent. Males demonstrated greater peak torque LSI in Ebert et al. [49], San Jose et al. [51], and Yasuda et al. [53] at every postoperative interval. Females demonstrated greater peak torque LSI in Otzel et al. [54] at 180°/s and in both Tampa Scale of Kinesiophobia (TSK) strata of Rips et al. [56]; San Jose’s isometric MVIC LSI showed no sex difference. Peak torque to bodyweight (Nm/kg) favored males in every comparison across all four studies reporting it. However, when those same absolute capacity values were re-expressed as a percentage of sex-specific normative data, the male advantage was not uniform. At 60°/s, females’ percentage of normative capacity exceeded or matched males’ in three of seven paired comparisons (Zore’s pre-pandemic control cohort, Zore’s 2022 cohort, and Rips’ greater -kinesiophobia [TSK > 37]), while males had the clear advantage in the remaining four. At 180°/s, males had the clear advantage as the percentage of normative capacity exceeded that of females in all four comparisons. Power in the form of rate of torque development was assessed in only two studies, in which San Jose et al. [51] found significantly lower isometric RTD LSI in females despite no significant isometric strength differences in the same cohort assessed. Kuenze et al. [55] found equivalent pre-exercise superimposed-burst torque between sexes that diverged only after a fatiguing bout following exercise (−1.45% males vs. −10.32% females, *p* = 0.02). Morphology was assessed by thigh circumference in a single study by Otzel et al. [54] that showed no sex difference within the ACLR cohort (*p* = 0.442) despite significant difference in the study’s healthy, uninjured controls (57 cm vs. 52 cm, *p* < 0.05).

Combining the outcomes assessed, the findings do not support a simple narrative that females have difficulty restoring quadriceps function compared to males after ACLR. Instead, whether a sex difference is detected and which sex it favors depends on the construct being measured and the testing velocity or task demand at which absolute capacity is assessed.

### 4.2. Overall Completeness and Application of Evidence

The evidence base that was assembled is narrow relative to the clinical importance of this question. Seven studies, in which one randomized controlled trial (RCT) is insufficient to fully characterize sex-based recovery across the diversity of graft types, sports, and rehab protocols that are encountered in practice. The coverage between the three predefined outcome domains was also uneven, in which quadriceps strength was addressed by all seven studies, true power/RTD by only one study, fatigue-induced torque decline (a related but distinct construct) by one additional study, and morphology by only one study that utilized a circumference measure rather than cross-sectional imaging or architectural ultrasound. The follow-up windows ranged from approximately three months to 5.5 years, which is a strength that captures both early and late recovery. However, this also means that findings from a three-month cohort (San Jose et al. [51]) and five-year cohort (Rips et al. [56]; Yasuda [53]) are being synthesized alongside one another as if they represent comparable points on a single trajectory when, in fact, postoperative physiology at these time points are substantially different. Applicability is also constrained due to participants being recreationally active or competitive pivoting-sport athletes, and not including sedentary individuals. None of the included studies reported menstrual cycle phase, hormonal status, or contraceptive use despite a plausible biological rationale [26] for hormonal effects on neuromuscular recovery, and this is a gap in the field that has not yet begun to close, which this review cannot fill in.

### 4.3. Quality of Evidence

Methodological quality was acceptable within study-design limitations based on the risk of bias assessed for the included studies. The instrumentation utilized in the studies was uniformly greater quality for isokinetic and isometric dynamometry, demonstrating intraclass correlation coefficients (ICC) of at least 0.74 across all instruments, with most exceeding 0.85. Despite the greater instrument-level reliability, the overall certainty level of evidence for any specific sex comparison is decreased, as a single study provides estimates regarding power and morphology. There was also inconsistency across studies for peak torque LSI, as well as the absence of any level I evidence with adequate blinding, which reduces the overall confidence that these effect estimates would be reproduced in a completely different sampled population. Applying this narrative approach outcome by outcome, using the GRADE domains of risk of bias, inconsistency, indirectness, and imprecision, no outcome in this review reaches moderate or high certainty; all fall in the low-to-very-low range. Peak torque limb symmetry index (LSI) was reported by five studies (Ebert et al. [49], San Jose et al. [51], Yasuda et al. [53], Otzel et al. [54], Rips et al. [56]). Certainty begins low because none are randomized comparisons of sex differences and most are retrospective cohorts; it is downgraded further for inconsistency, since the direction of the sex difference varied by study and by testing velocity, and for indirectness, since LSI conflates involved-limb recovery with uninvolved-limb detraining. With per-sex subgroup sizes frequently under 30, imprecision is a further concern. Overall certainty for peak torque LSI: very low. Peak torque normalized to body weight (absolute-capacity) was reported by four studies (Kuenze et al. [55], Otzel et al. [54], Zore et al. [52], Rips et al. [56]). Certainty begins low for the same design reasons; it is not further downgraded for inconsistency, since the direction favoring males was consistent across comparisons, but it is downgraded for indirectness. The overall certainty is then characterized as low for peak torque to body weight. The normative-referenced percentage comparisons are a secondary, exploratory analysis constructed for this review rather than an outcome directly reported by the primary studies. The certainty begins low and is substantially downgraded for indirectness, since the ACLR cohorts were compared against externally sourced normative datasets that differ in age, training status, and testing context, and further downgraded for imprecision because confidence intervals could not be calculated for the derived percentages. Overall certainty for the normative-referenced values secondary analysis is rated as: very low. Quadriceps power (rate of torque development) rests on a single study (San Jose et al. [51]), fatigue-induced torque decline on a single study (Kuenze et al. [55]), and morphology on a single study (Otzel et al. [54]). For each, certainty begins low and is downgraded for serious imprecision because a single small study cannot establish precision or replicability, with morphology additionally downgraded for indirectness because circumference is only an indirect surrogate for the muscle properties of interest. Overall certainty for each of these three outcomes: very low. Taken together, every outcome domain in this review carries low or very low certainty, which is the primary reason its conclusions are framed as hypothesis-generating throughout rather than as established findings.

### 4.4. Potential Biases in the Review Process

There are a few potential review biases that should be acknowledged. Publication bias is plausible in studies finding no sex differences in quadriceps outcomes may be less likely to be written than studies reporting a contrast. This would then inflate the frequency of significant sex effects in the literature relative to the underlying frequency of sex comparison outcomes. The restriction to English-only language full-text publications may have excluded valuable non-English-language studies. It is important to note that most included studies did not rely on sex-based quadriceps comparisons as the primary aim. The extraction relied on subgroup or secondary analysis within the studies. The reviewer judgment in eligibility screening, although performed independently by two reviewers, remains a subjective process and should take into account potential bias.

### 4.5. Interpretation of Findings

A central interpretive issue running through the literature is that the peak torque limb symmetry index treats the uninvolved limb as a proxy for the patient’s preinjury state. Cross-education effects, as well as disuse during early rehabilitation periods and altered loading patterns, produce measurable strength loss in the contralateral limb within weeks of surgery. Wellsandt et al. [18] demonstrated that using the uninvolved limb as a recovery reference systematically overestimates true symmetry by 8–15%. Under the current framework, peak torque LSI differences between sexes is ambiguous as it could reflect genuinely worse involved limb recovery in one sex, or greater uninvolved-limb detraining in one sex, or a baseline sex difference in strength that may produce a different ratio when both limbs have lost a portion of total strength capacity. None of the four studies reporting peak torque LSI reported a preoperative or age/sex matched normative value alongside it; ambiguity cannot be resolved within the available data. With the ambiguity acknowledged above, following the trends in the twelve peak torque LSI values, we do see females consistently having decreased peak torque LSI compared to males, except for Otzel’s 180°/s comparison. Female peak torque LSI reached the commonly used 90% RTS threshold in only two of twelve instances (Otzel’s 60°/s and 180°/s within-limb comparisons, 91% and 93%), both drawn from the same small cohort (13 females). In practical terms, females would only clear the 90% LSI threshold when in a small sample cohort, while male patients cleared it across a broader and more independent set of studies and conditions. This does not establish that females recover peak torque symmetry worse in a biological sense. However, it does mean that a fixed 90% peak torque LSI cutoff applied without sex-specific calibration will flag female patients as not ready far more often than males, regardless of whether the underlying recovery is worse. This descriptive pattern shows that females clear the threshold less often; it does not by itself show that 90% is an inappropriate or poorly calibrated threshold specifically for females. Establishing that would require validating the threshold against a meaningful outcome such as successful return to sport or subsequent reinjury, which none of the included studies examined, and so this distinction between statistical pattern and threshold validity should be kept separate.

The intuitive alternative to peak torque LSI is comparing peak torque to bodyweight between males and females is not a solution to the healthy limb problem because it then introduces a different one. A large, well-documented sex difference in quadriceps strength relative to body weight exists in the general uninjured population, with normative female values typically running 60–75% of male values at a given testing velocity [19,36,37]. This review’s own data illustrates the same point, independent of any injury, in which Otzel et al. [54] demonstrated uninjured control participants showed significantly greater thigh circumference in males than females. The finding that ACLR males produced greater peak torque to body weight than ACLR females could mean recovery genuinely differs by sex, or mean both sexes recovered the same proportion of their own normal strength, just beginning from different starting points. The peak torque to body weight comparisons cannot distinguish these two possibilities and every prior sex comparison study that reports only raw Nm/kg values is vulnerable to overinterpretation of a pre-existing population difference as a recovery deficit.

When the studies in the review were compared to normative reference values re-expressed as a percentage of the sex-specific normative values, it becomes a lot more nuanced than “males recovered better”. At the slower, more strength-dominant 60°/s velocity, females reached or exceeded their own normative capacity relative to males in three of eight paired comparisons: Zore’s pre-pandemic control cohort (78.9% female vs. 74.6% male), Zore’s 2022 surgical-year cohort (80.2% vs. 74.1%), and Rips’ higher-kinesiophobia stratum (82.8% vs. 79.3%, non-significant). In the remaining five 60°/s comparisons, males’ percentage of normative capacity was greater, but by margins considerably smaller than what the raw Nm/kg gap alone would suggest. However, at the faster rate dependent speed of 180°/s, males exceeded females percentage of normative comparison in all four available comparisons (Otzel involved 73.8% vs. 52.0%; Otzel uninvolved 81.7% vs. 56.4%; Rips TSK > 37 57.5% vs. 52.0%; Rips TSK ≤ 37 62.3% vs. 50.0%). This velocity-dependent pattern is consistent both mechanistically and empirically with this review’s findings regarding power. The two studies assessing quadriceps power (San Jose et al. [51]; Kuenze et al. [55]) identified a female disadvantage that was absent at rest and under low velocity compared to fatigue and faster velocity. A consistent, generalizable female recovery deficit is not well-supported at slower, sustained tasks; however, a more defensible deficit emerges specifically with faster contraction velocities, as well as under fatigue or explosive demands.

#### 4.5.1. Alternative Interpretations of Data

This interpretation is not the only one that the data in this study can support. An alternative reading of the 60°/s-versus-180°/s divergence is that it reflects graft-type or sample-composition confounding rather than a true velocity-dependent sex effect. Rips et al. [56] had a substantial sex-by-graft imbalance favoring bone–patellar tendon–bone (BTB) autografts in males. BTB grafts carry their own well-documented research that early quadriceps strength, independent of sex, could affect the two independent testing velocities if experiencing anterior knee pain, and is the rate-limiting factor at greater velocity. Ebert et al. [49] identified a significant sex-by-graft interaction in which females that had quadriceps tendon autografts showed the least peak torque LSI of any of the subgroups. This indicates that sex and graft choices are not independent variables in the literature. The velocity-dependent neuromuscular sex difference and graft-driven confounding variables remain important factors that the review cannot fully determine. It is also possible that symmetry-based measures such as the LSI behave differently across testing velocities independent of any true sex-based recovery difference, because bilateral and unilateral force-generating capacity are not simply proportional to one another as contraction speed increases [59]. This review cannot separate that measurement-level explanation from a genuine physiological sex-by-velocity interaction.

#### 4.5.2. Agreement and Disagreement with Prior Literature

The overall impression that female patients experience less favorable quadriceps outcomes after ACLR is broadly consistent with prior work. Ageberg et al. [27] reported poorer patient-reported knee function and quality of life in females using the Swedish registry data. This is also consistent with Kim et al. [28], who reported greater residual quadriceps weakness in females at one year. Where this review diverges from existing results is that it does not treat recovery-referenced peak torque LSI and absolute capacity as interchangeable or reinforcing evidence of the same sex-based recovery difference. These measures assess different aspects of quadriceps function and utilize sex-specific normative values, because then the male advantage became less consistent and varied between testing velocities. This means that the findings of males producing greater Nm/kg values across studies do not necessarily indicate that males recovered a greater proportion of their expected quadriceps capacity. The distinction is clinically important because it may explain why previous narrative summaries have characterized female disadvantage as more uniform in nature than the primary data supports when compared to sex-specific metrics, as well as testing velocity.

This is the first systematic review to isolate quadriceps recovery following ACLR in an adult-only population. Restricting it to adults removes pediatric skeletal maturation as a confounding variable that has complicated the interpretation of prior sex comparison literature. The review’s principal conceptual contribution is methodological by distinguishing recovery reference and absolute capacity outcomes as answering different clinical questions and then comparing them with the available absolute capacity data as a percentage of sex-specific normative values rather than just comparing the raw values with each other. The re-analysis is new evidence that changes the conclusion from males showing a consistent absolute-strength advantage to the absolute-capacity sex difference being velocity-dependent, present at 180°/s, and largely absent or reversed at 60°/s once normative capacity is accounted for. This provides more precise evidence in support of existing proposals that fatigue and rate-sensitive neuromuscular function, rather than maximal strength under ideal testing conditions, is where the true sex-based recovery differences after ACLR are.

### 4.6. Significance of Findings

For patients, these findings argue against interpreting a raw between sex strength comparison or a single peak torque LSI value as a verdict on recovery quality and end-all be-all for return-to-sport decision-making. A female patient’s peak torque to body weight will be predictably decreased than a male patient’s, even after successful rehabilitation programming. That difference should not be used to suggest she has recovered less well. Where the genuine deficit does exist is in more velocity and explosive specific tasks with fatigue. This then has direct relevance to how confident a patient and clinician can be heading into cutting, pivoting, and sport-specific movements to minimize the greater retear rates present among females.

For clinicians, this review supports referencing absolute strength testing against sex matched normative data or against the involved limb’s own preoperative value, where available. It also supports building greater velocity as well as rate of torque development tasks under fatigue-related conditions into the RTS battery rather than relying on a single well-rested maximum effort contraction or plyometrics. The patient will have to be used to greater demands when returning to performance, therefore it is essential that we test them under similar conditions. The return-to-sport clearance window should remain criteria-based rather than tied to the usual 9–12-month windows that are present in most surgical protocols. This is due to a recovery referenced gap that tends to be open early in the postoperative course based on the literature in this study (Yasuda [53]: 79% vs. 78% pre-operatively to 51% vs. 33% by 3 months, *p* < 0.001) and does not reliably close with time alone.

The registries and clinical databases with the structure to track ACLR outcomes are well positioned to require that strength and power outcomes be reported separately by sex and referenced against normative data, which would advance the field’s ability to detect and confirm sex-based recovery differences. This study showed that there are not many studies, if any, that look at sex-specific outcomes as a primary outcome for adults.

### 4.7. Limitations of the Review

The limitations of this review are the smaller number of eligible studies being n = 7, which limits the statistical power and ability to complete meta-analysis. The decreased amount of studies looking at power and morphology in adults is also a limitation that must be regarded as preliminary and hypothesis-generating for future studies. There were no included studies that were Level I evidence with adequate blinding, as well as a decreased number of randomized controlled trials. The populations were predominantly athletic and recreationally active, which limits the ability to generalize to the sedentary population. Graft type also varied substantially across and within studies and was not consistently controlled for, which introduces heterogeneity. Graft type was also unevenly distributed by sex in some studies (e.g., Ebert et al. [49]; Rips et al. [56]), this review cannot fully separate a true velocity-dependent sex effect from graft-related or sample-composition confounding, and the velocity- and fatigue-dependent pattern described in Section 4.5 should be read as a hypothesis rather than a demonstrated finding. The restriction to English-language publications is a further limitation, particularly given that only seven of 1411 screened records ultimately met all eligibility criteria; relevant non-English evidence may have been missed. Outcome assessments also varied in general protocol, velocity, and device, which then increases between-study heterogeneity. The single study that looked at morphology relied on circumference rather than imaging to look at cross-sectional area (CSA), which is important to note.

### 4.8. Recommendations for Future Research

The future cohort studies should prospectively stratify by sex as a primary analysis rather than a subgroup, as much of the literature is focused on men rather than women. Randomized controlled trials for adults are needed to raise the level of evidence in this space beyond the single RCT currently available. The studies should also report absolute strength as a percentage of the sex matched normative values using the published reference data sets (Van Melick et al. [19], Barrué-Belou et al. [36], or Šarabon et al. [37]) rather than the raw Nm/kg values. There should also be an inclusion of preinjury reference values for peak torque LSI to get true values on recovery. Quadriceps morphology should also be assessed with proper imaging techniques. Future ACLR protocols should build in the rate of torque development testing under fatigue conditions. It is also suggested to utilize more adult-only populations, as this area is extremely thin for an important topic. It is also worthy to note the lack of studies that focus on females, and that a lot of the data relies on male-only populations of interest.

## 5. Conclusions

This review is the first to synthesize the evidence examining sex-based differences in quadriceps strength, power, and morphology among adults following primary unilateral ACL reconstruction while minimizing the influence of pediatric skeletal maturation. Symmetry-referenced outcomes for peak torque were inconsistent across studies, and female peak torque limb symmetry index rarely reached the 90% peak torque LSI for return to performance, while males did so more often. Peak torque normalized to body weight favored males in every study, but this comparison is confounded by non-injury-related sex differences in strength. When re-expressed as a percentage of each sex’s normative capacity, the females matched or exceeded males’ relative recovery in several 60°/s comparisons, while males held a consistent advantage at 180°/s. Quadriceps power (RTD) was assessed in only one study and showed a female disadvantage; a second, related study found a fatigue-induced decline in absolute torque that was greater in females, a distinct but complementary finding. The review’s central findings are that a female recovery deficit is not well supported for slower, maximal-effort strength, and that a pattern consistent with a greater deficit at higher velocities and under fatigue is present in the data but rests on very few studies with important confounding by graft type and sample composition; this pattern should be treated as hypothesis-generating rather than established. Clinically, this argues against comparing raw strength values across sexes or relying on fixed peak torque LSI and in favor of referencing against sex and age normative data. Whether incorporating fatigue- and power-sensitive testing into return-to-sport criteria improves outcomes is a hypothesis raised by this review rather than a conclusion it establishes. Given the small number of eligible studies and the absence of Level I literature, these conclusions should be treated as hypothesis generation with future prospective sex stratified research to confirm them.

## Figures and Tables

**Figure 2:**
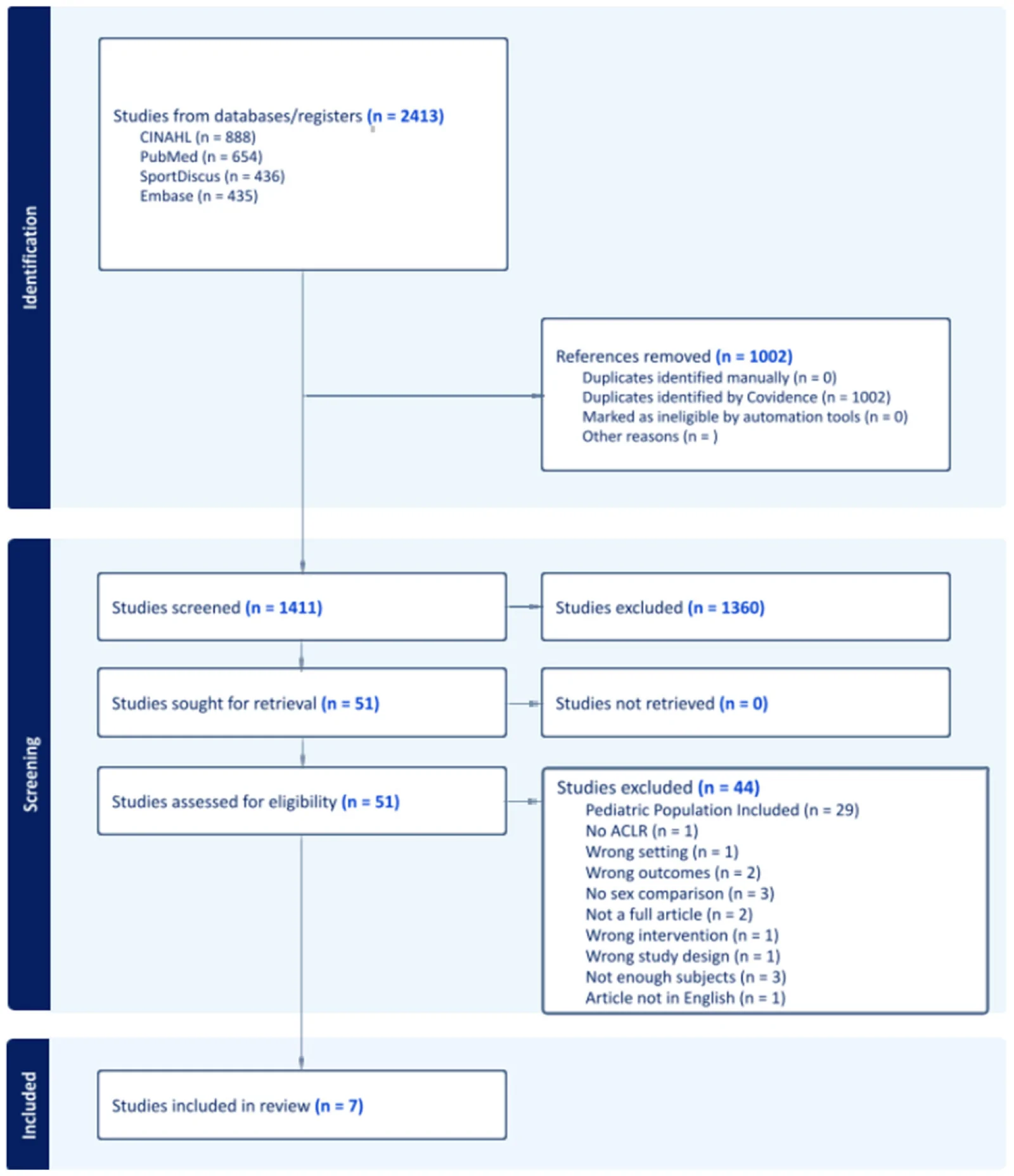
The PRISMA flow diagram.

**Figure 3:**
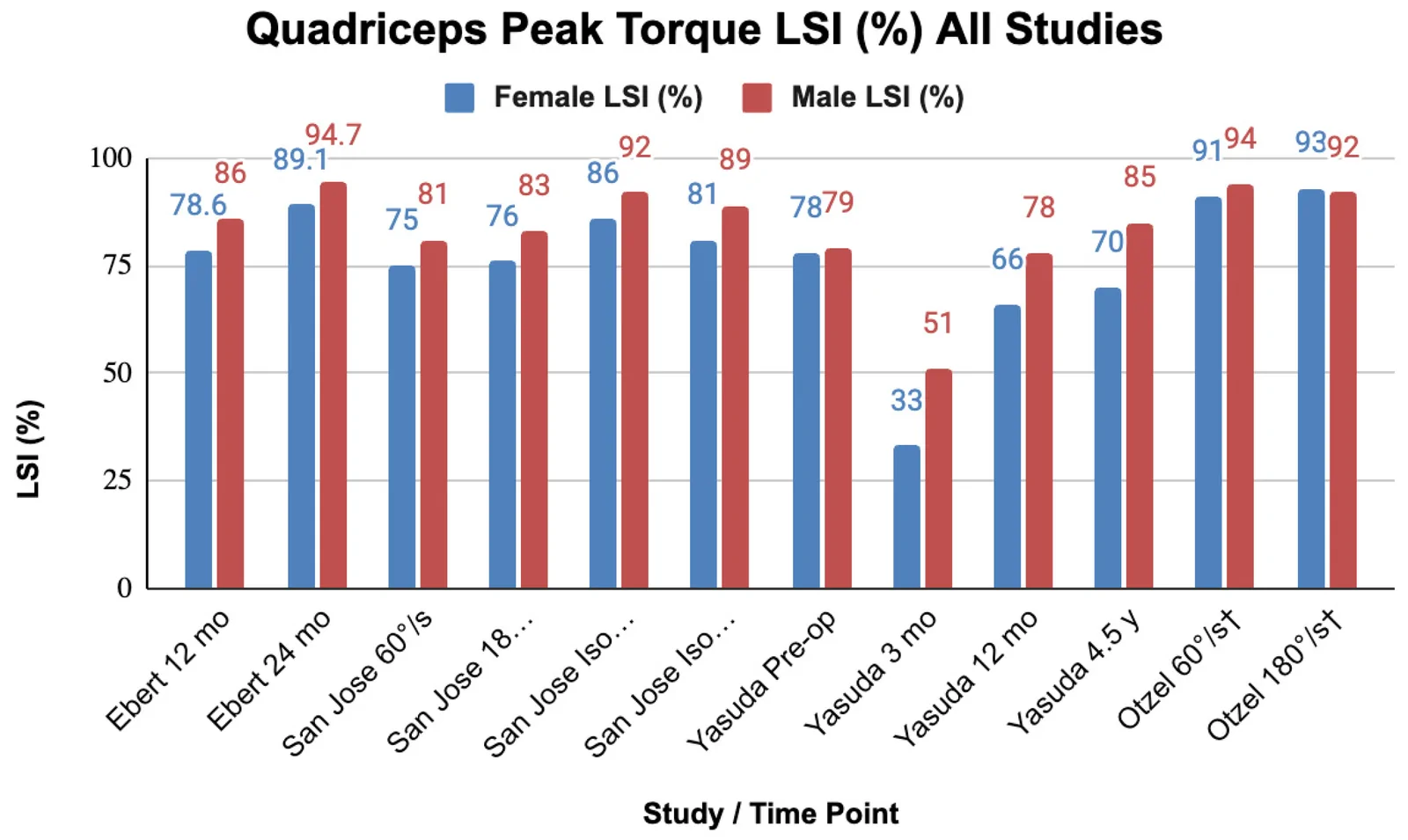
Quadriceps peak torque LSI (%), all studies.

**Figure 4:**
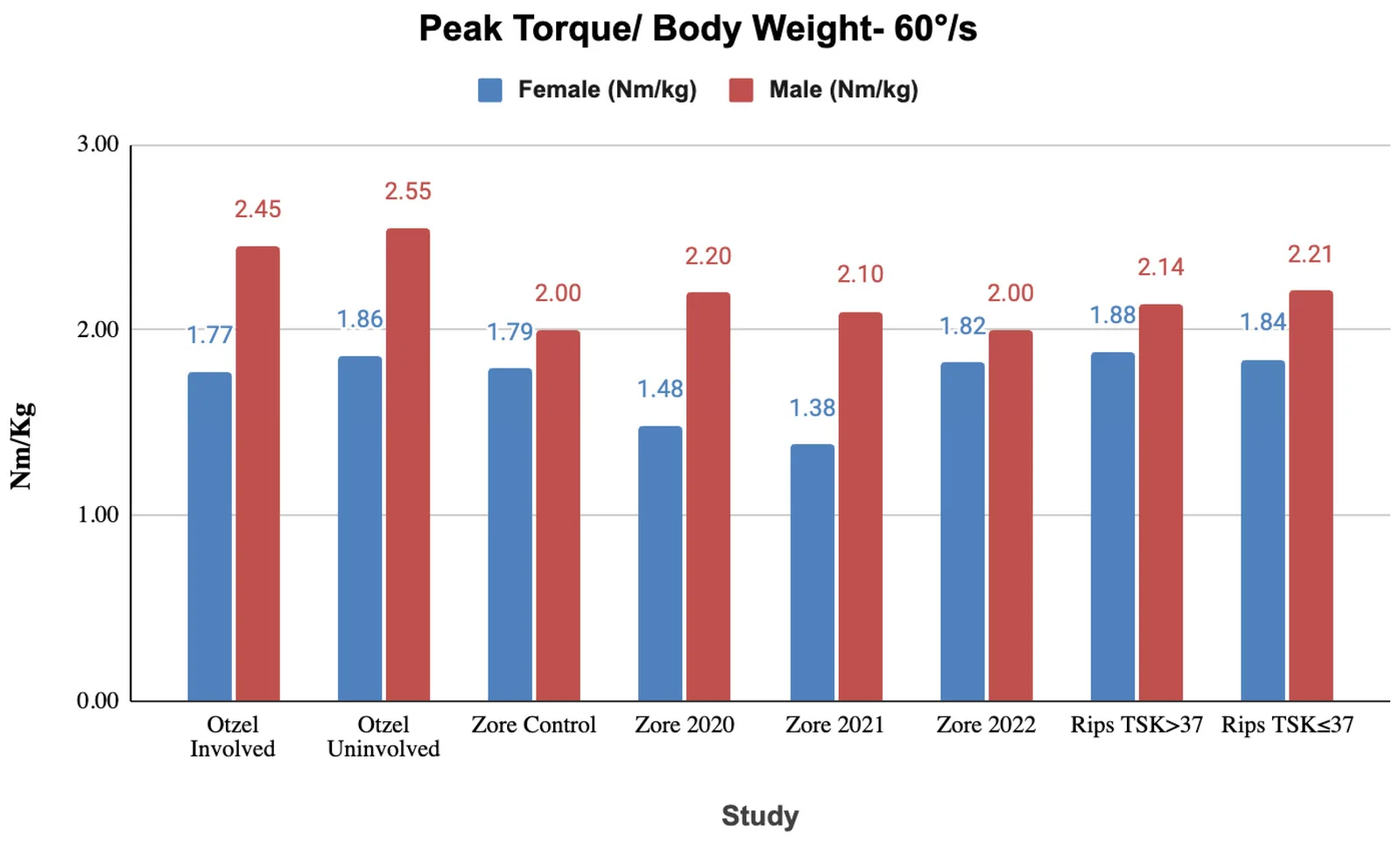
Peak torque to body weight, 60°/s.

**Figure 5:**
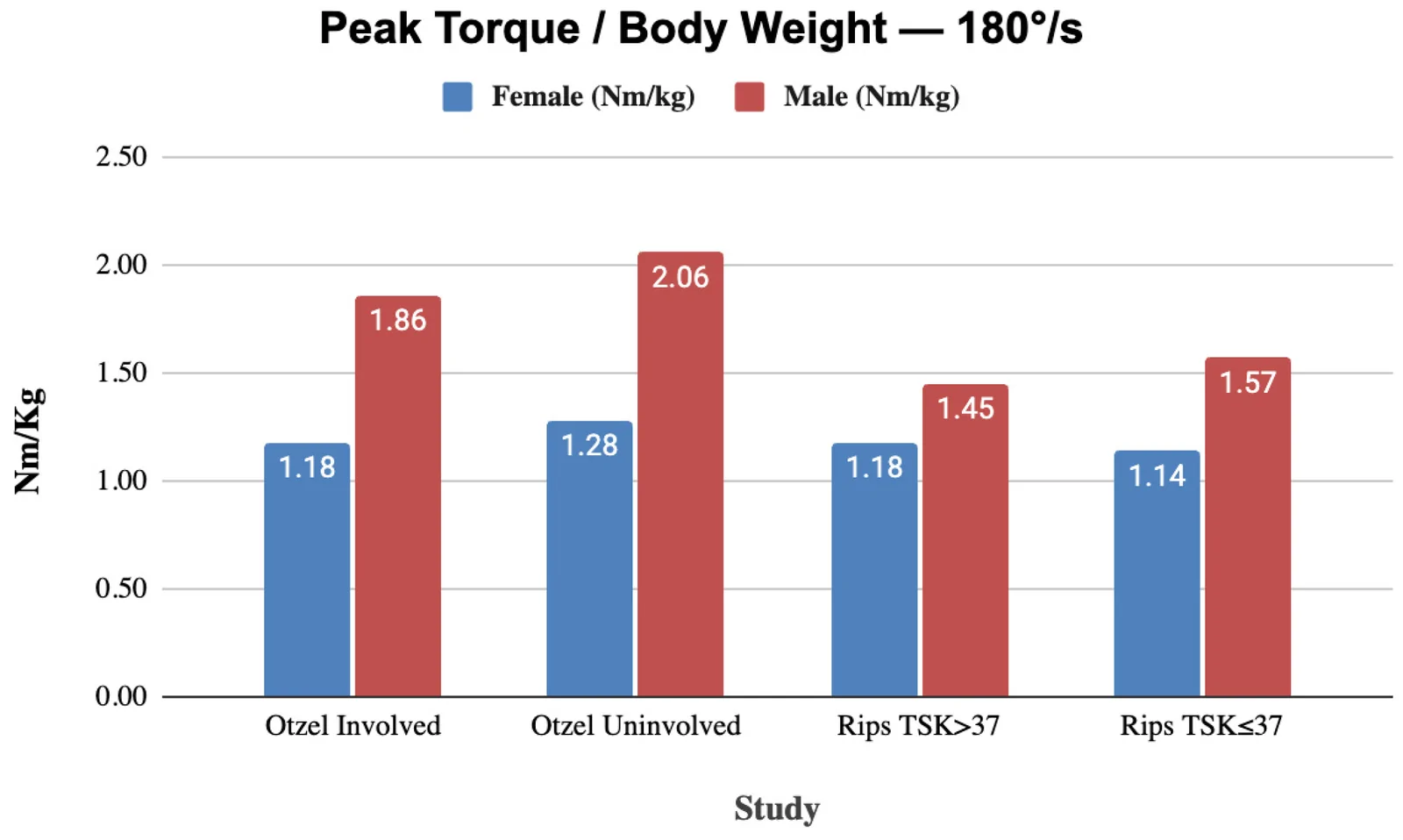
Peak torque to body weight, 180°/s.

**Figure 6:**
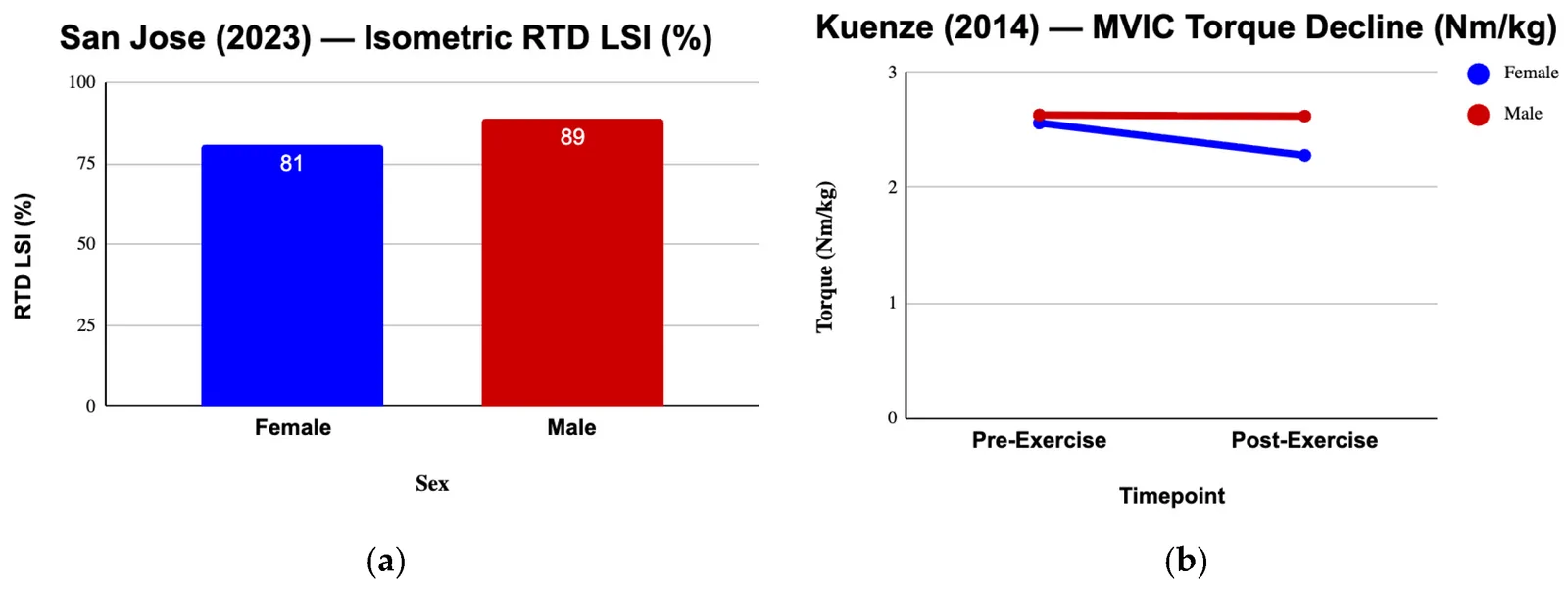
Sex comparisons regarding power. (**a**) San Jose (2023) isometric RTD LSI (%) by sex. (**b**) Kuenze (2014) MVIC torque decline (Nm/kg) pre- to post-exercise, by sex.

**Table 1:** PECO question and study design inclusion and exclusion criteria.

Question Component	Inclusion Criteria	Exclusion Criteria
Population	• Population: Adults (≥18 years) who underwent primary unilateral ACL reconstruction.	• Revision ACLR • Multi-ligament reconstruction • Pediatric populations
Exposure	• Studies including both male and female participants with sex-specific data reported.	• Single-sex studies
Comparison	• Studies reporting sex-specific quadriceps outcomes or direct comparisons between male and female participants.	• Studies that do not report quadriceps outcomes separately by sex, and where sex-based comparison cannot be determined.
Outcome	• Studies reporting at least ONE objective measure of quadriceps morphology, strength, or power: Quadriceps Strength (isokinetic/isometric peak torque, peak torque to body weight); Quadriceps Power/Explosive Capacity (rate of torque development [RTD], power or impulse during knee extension or functional testing); Quadriceps Morphology (CSA, muscle thickness, circumferential volume, fiber CSA via imaging or biopsy). Measurements must be obtained using objective instrumented testing or validated imaging techniques.	• Studies using non-objective or insufficiently valid quadriceps strength measures (e.g., MMT alone, subjective strength grading) • Studies not reporting outcomes related to quadriceps morphology, strength, or power.
Study Design	• RCTs; prospective/retrospective cohort studies; case–control studies; cross-sectional studies; studies with ≥10 participants in the smaller sex group; studies published in English or with an accessible translation.	• Case reports • Conference abstracts without full-text publication • Studies with insufficient data to extract sex-specific quadriceps outcomes

**Table 2:** Level of evidence, modified from the Oxford Centre of Evidence-Based Medicine (OCEBM).

Level of Evidence	Study Characteristics
I	Evidence obtained from high-quality randomized controlled trials, prospective studies, or diagnostic studies
II	Evidence obtained from lesser quality randomized control trials, prospective studies, or diagnostic studies (i.e., improper randomization, no blinding, less than 80% follow-up)
III	Case–control, cohort, or retrospective studies
IV	Case series
V	Expert opinion

Note: OCEBM Levels of Evidence Working Group. The Oxford Levels of Evidence 2. Oxford Centre for Evidence-Based Medicine [43].

**Table 3:** Level of certainty (GRADE criteria for levels of certainty for evidence).

Certainty of Evidence	Study Characteristics
High	We are very confident that the true effect lies close to that of the estimate of the effect.
Moderate	We are moderately confident in the estimate of effect: the true effect is likely to be close to the estimate of effect, but possibility to be substantially different.
Low	Our confidence in the effect is limited: the true effect may be substantially different from the estimate of the effect.
Very Low	We have very little confidence in the effect estimate: the true effect is likely to be substantially different from the estimate of effect.

Note: Schünemann, H.J. Interpreting GRADE’s levels of certainty or quality of the evidence [44].

**Table 6:** (**A**). Summary of risk of bias for randomized controlled trials using the PEDro scale. (**B**). Summary of risk-of-bias assessment for cohort studies using JBI. (**C**). Summary of risk-of-bias assessment for cross-sectional studies using JBI. (**D**). Summary of risk-of-bias assessment for quasi-experimental studies using JBI. (**E**). Summary of risk-of-bias assessment for the case series using JBI.

Study Author (Year)	1	2	3	4	5	6	7	8	9	10	11	Score
**A**												
Ebert et al. [42]	✓	✓	✗	✓	✗	✗	✗	✓	✗	✓	✓	5 FAIR
**B**												
San Jose et al. [45]	✓	✓	✓	✓	✗	✓	✓	✓	✓	✗	✓	8 Low Risk
Zore et al. [46]	✓	✓	✓	✓	✓	✓	✓	✓	✓	✓	✓	10 Low Risk
Yasuda et al. [47]	✓	✓	✓	✓	✗	✓	✓	✓	✗	✗	✓	7 Moderate Risk
**C**												
Otzel et al. [44]	✓	✓	✓	✓	✗	✓	✓	✓	7 Low Risk			
**D**												
Kuenze et al. [43]	✓	✗	✓	✓	✓	✓	✓	✓	✓	8 Low Risk		
**E**												
Rips et al. [48]	✓	✓	✓	✗	✗	✓	✓	✓	✓	✓	✓	8 Low Risk

Note: (**A**). Scores 9–10 classified as ‘excellent’, 6–8 as ‘good’, 4–5 as ‘fair’, and scores below 4 as ‘poor quality’. Note: (**B**). Scores of 8–10 = ‘low risk’, 5–7 = ‘moderate risk’, 0–4 = ‘high risk’. Note: (**C**). Scores of 7–8 = ‘low risk’, 5–6 = ‘moderate risk’, 0–4 = ‘high risk’. Note: (**D**). Scores of 8–9 = ‘low risk’, 5–7 = ‘moderate risk’, 0–4 = ‘high risk’. Note: (**E**). Scores of 8–10 = ‘low risk’, 5–7 = ‘moderate risk’, 0–4 = ‘high risk’.

**Figure A1:**
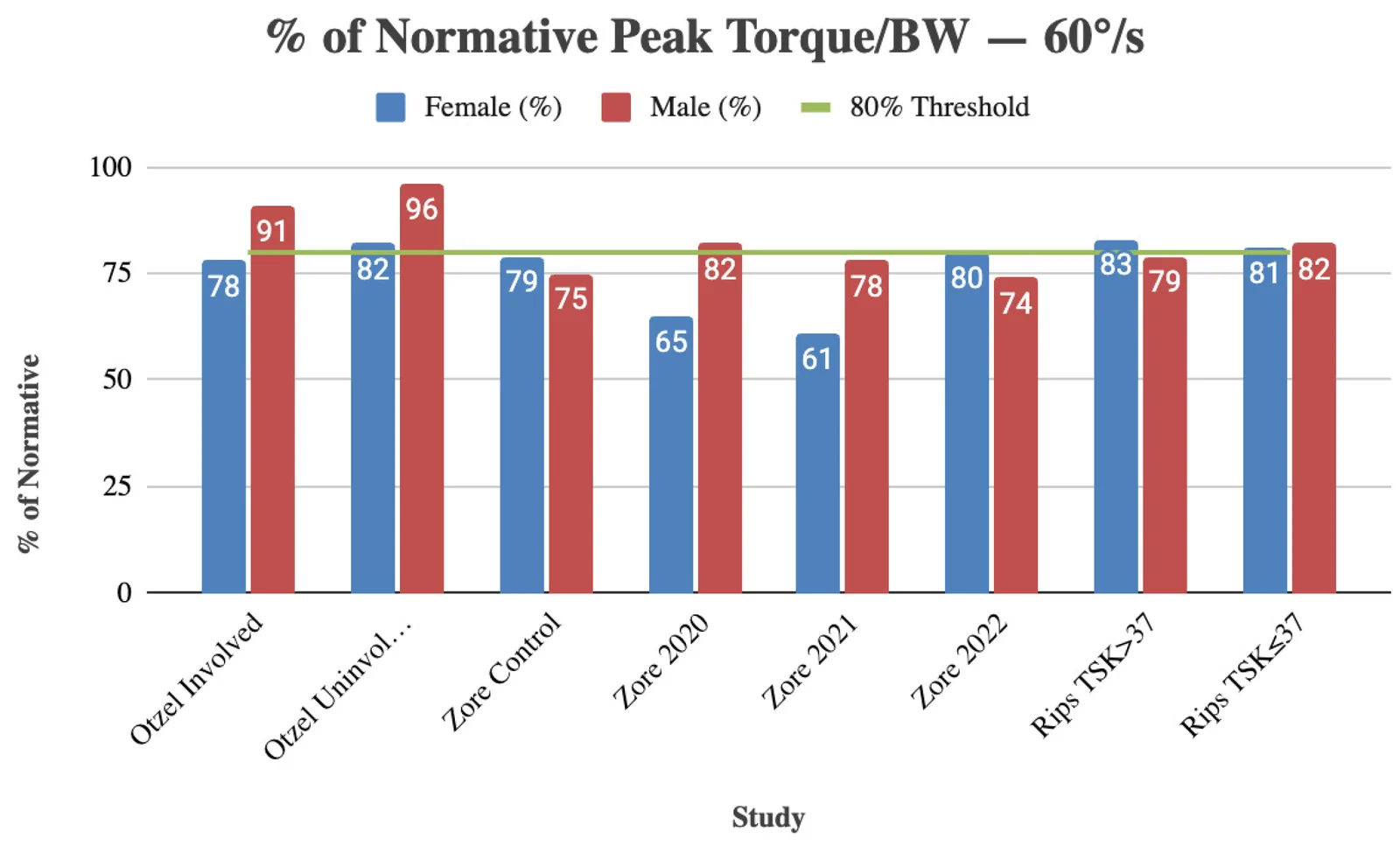
% of normative peak torque/body weight—60°/s.

**Figure A2:**
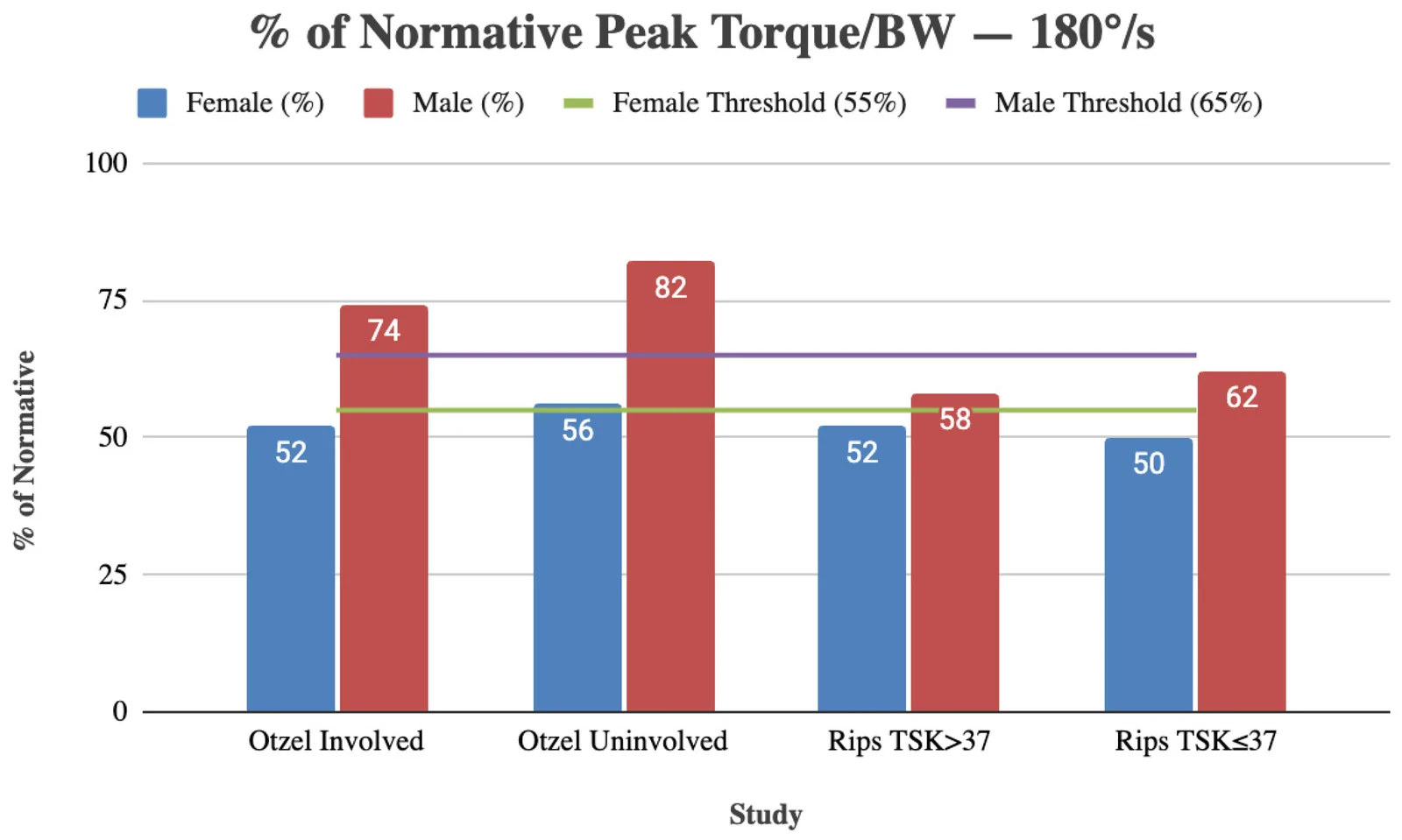
% of normative peak torque/body weight—180°/s.

## Data Availability

No new data were created or analyzed in this study. All data extracted for this review are available within the article and its appendices, or from the original publications cited in the reference list. The review protocol is registered with PROSPERO (Registration No. CRD420261338598).

## Abbreviations

The following abbreviations are used in this manuscript:

ACLR	Anterior cruciate ligament reconstruction
ACL	Anterior cruciate ligament
LSI	Limb symmetry index
RTD	Rate of torque development
RFD	Rate of force development
RTS	Return to sport
MVC	Maximal voluntary contraction
MVIC	Maximal voluntary isometric contraction
CSA	Cross-sectional area
Q-AI	Quadriceps asymmetry index
TSK	Tampa Scale of Kinesiophobia
JBI	Joanna Briggs Institute
PEDro	Physiotherapy Evidence Database
PRISMA	Preferred Reporting Items for Systematic Reviews and Meta-Analyses
PECO	Population, Exposure, Comparison, Outcome
OCEBM	Oxford Centre for Evidence-Based Medicine
GRADE	Grading of Recommendations Assessment, Development and Evaluation
BTB	Bone–patellar tendon–bone
HT	Hamstring tendon
PT	Patellar tendon
QT	Quadriceps tendon
ICC	Intraclass correlation coefficient

## Appendix A. Search Strategies

**PubMed**

((“Anterior Cruciate Ligament Reconstruction”[Majr] OR “Anterior Cruciate Ligament Reconstruction/rehabilitation”[Mesh] OR “Anterior Cruciate Ligament Injuries”[Majr] OR “Anterior Cruciate Ligament Injuries/surgery”[Mesh] OR “Anterior Cruciate Ligament Injuries/physiopathology”[Mesh] OR “Anterior cruciate ligament”[Title/Abstract] OR “ACL”[Title/Abstract] OR “ACLR”[Title/Abstract]) AND ((“Quadriceps Muscle”[Mesh] OR “Quadriceps Muscle/physiology”[Mesh] OR “Quadriceps Muscle/physiopathology”[Mesh] OR quadriceps[Title/Abstract] OR “quadriceps function”[Title/Abstract:~2] OR “quadriceps size”[Title/Abstract:~2] OR “quadriceps power”[Title/Abstract:~2] OR “quadriceps strength”[Title/Abstract:~2] OR “quadriceps performance”[Title/Abstract:~2]) AND (((“Muscle Strength”[Mesh]) OR “Muscle Strength/physiology”[Mesh] OR “Muscle Weakness”[Mesh] OR “Muscle Weakness/etiology”[Mesh] OR “Muscle Weakness/physiopathology”[Mesh] OR “Muscle Weakness/rehabilitation”[Mesh] OR “Muscle Fibers, Skeletal/pathology”[Mesh] OR “Muscular Atrophy”[Mesh] OR “Muscular Atrophy/pathology”[Mesh] OR “Muscular Atrophy/physiopathology”[Mesh] OR “Muscular Atrophy/rehabilitation”[Mesh] OR “Muscle Contraction”[Mesh] OR “Torque”[Mesh] OR strength[Title/Abstract] OR “muscle strength”[Title/Abstract] OR torque[Title/Abstract] OR “peak torque”[Title/Abstract] OR isokinetic[Title/Abstract] OR dynamometr*[Title/Abstract] OR power[Title/Abstract] OR “muscle power”[Title/Abstract] OR “rate of torque development”[Title/Abstract] OR “RTD”[Title/Abstract] OR “cross-sectional area”[Title/Abstract] OR “CSA”[Title/Abstract] OR “muscle size”[Title/Abstract] OR volume[Title/Abstract] OR girth[Title/Abstract] OR contraction[Title/Abstract] OR hypertroph*[Title/Abstract] OR atroph*[Title/Abstract] OR “fiber type”[Title/Abstract] OR “fibre type”[Title/Abstract]))) AND ((“Sex Factors”[Mesh] OR “Sex Differences”[Mesh]) OR (“sex differences”[Title/Abstract:~3] OR “sex based”[Title/Abstract:~3] OR “sex related”[Title/Abstract:~3] OR “sex characteristics”[Title/Abstract:~3] OR “gender differences”[Title/Abstract:~3] OR “gender based”[Title/Abstract:~3] OR “gender related”[Title/Abstract:~3] OR male*[Title/Abstract] OR female*[Title/Abstract] OR man[Title/Abstract] OR men[Title/Abstract] OR Woman[Title/Abstract] OR women[Title/Abstract]))

**Results: 653**
**Embase**

(‘anterior cruciate ligament reconstruction’/exp/mj OR ‘anterior cruciate ligament reconstruction’/exp/dm_rh OR ‘anterior cruciate ligament injury’/exp/mj OR ‘anterior cruciate ligament injury’/exp/dm_su OR ‘anterior cruciate ligament’:ti,ab,kw OR ‘acl’:ti,ab,kw OR ‘aclr’:ti,ab,kw) AND (‘quadriceps femoris muscle’/exp OR ‘quadriceps’:ti,ab,kw OR (‘quadriceps’ NEAR/3 ‘function’):ti,ab,kw OR (‘quadriceps’ NEAR/3 ‘size’):ti,ab,kw OR (‘quadriceps’ NEAR/3 ‘power’):ti,ab,kw OR (‘quadriceps’ NEAR/3 ‘strength’):ti,ab,kw OR (‘quadriceps’ NEAR/3 ‘performance’):ti,ab,kw) AND (‘muscle strength’/exp OR ‘muscle weakness’/exp/mj OR ‘muscle weakness’/exp/dm_et OR ‘muscle weakness’/exp/dm_rh OR ‘muscle atrophy’/exp OR ‘muscle atrophy’/exp/dm_rh OR ‘muscle contraction’/exp OR ‘torque’/exp OR ‘strength’:ti,ab,kw OR ‘muscle strength’:ti,ab,kw OR ‘limb symmetry index’:ti,ab,kw OR ‘torque’:ti,ab,kw OR ‘peak torque’:ti,ab,kw OR ‘isokinetic’:ti,ab,kw OR ‘dynamometr*’:ti,ab,kw OR ‘power’:ti,ab,kw OR ‘muscle power’:ti,ab,kw OR ‘rate of torque development’:ti,ab,kw OR ‘rtd’:ti,ab,kw OR ‘cross-sectional area’:ti,ab,kw OR ‘csa’:ti,ab,kw OR ‘muscle size’:ti,ab,kw OR ‘volume’:ti,ab,kw OR ‘girth’:ti,ab,kw OR ‘contraction’:ti,ab,kw OR ‘hypertroph*’:ti,ab,kw OR ‘atroph*’:ti,ab,kw OR ‘fiber type’:ti,ab,kw OR ‘fibre type’:ti,ab,kw) AND (‘sex factor’/exp OR ‘sex difference’/exp OR (‘sex’ NEAR/4 ‘differences’):ti,ab,kw OR (‘sex’ NEAR/4 ‘based’):ti,ab,kw OR (‘sex’ NEAR/4 ‘related’):ti,ab,kw OR (‘sex’ NEAR/4 ‘characteristics’):ti,ab,kw OR (‘gender’ NEAR/4 ‘differences’):ti,ab,kw OR (‘gender’ NEAR/4 ‘based’):ti,ab,kw OR (‘gender’ NEAR/4 ‘related’):ti,ab,kw OR ‘male*’:ti,ab,kw OR ‘female*’:ti,ab,kw OR ‘man’:ti,ab,kw OR ‘men’:ti,ab,kw OR ‘woman’:ti,ab,kw OR ‘women’:ti,ab,kw)

**Results: 435**
**CINAHL**

(MM “Anterior Cruciate Ligament Reconstruction” OR MH “Anterior Cruciate Ligament Reconstruction/RH” OR MM “Anterior Cruciate Ligament Injuries” OR MH “Anterior Cruciate Ligament Injuries/SU/PP” OR SU (“Anterior cruciate ligament” OR “ACL” OR “ACLR”)) AND (MH “Quadriceps Muscles” OR MH “Quadriceps Muscles/PP/PH” OR SU (quadriceps OR (quadriceps N3 (function OR size OR power OR strength OR performance)))) AND (MH “Muscle Strength” OR MH “Muscle Strength/PH” OR MH “Muscle Weakness” OR MH “Muscle Weakness/PP/RH” OR MH “Muscle Fibers/PA” OR MH “Muscular Atrophy” OR MH “Muscular Atrophy/PA/PP/RH” OR MH “Muscle Contraction” OR MH “Torque” OR SU (strength OR “muscle strength” OR “limb symmetry index” OR torque OR “peak torque” OR isokinetic OR dynamometr* OR power OR “muscle power” OR “rate of torque development” OR “RTD” OR “cross-sectional area” OR “CSA” OR “muscle N3 size” OR volume OR girth OR contraction OR hypertroph* OR atroph* OR (fiber N3 type) OR (fibre N3 type))) AND (MH “Sex Factors” OR SU ((sex N3 differences) OR (sex N3 based) OR (sex N3 related) OR (sex N3 characteristics) OR (gender N3 differences) OR (gender N3 based) OR (gender N3 related) OR male* OR female* OR man OR men OR Woman OR women))

**Results: 888**
**SPORTDiscus**

((DE “ANTERIOR cruciate ligament” OR DE “ANTERIOR cruciate ligament injuries” OR DE “ANTERIOR cruciate ligament surgery”) OR SU (“Anterior cruciate ligament” OR “ACL” OR “ACLR”)) AND ((DE “QUADRICEPS muscle” OR DE “QUADRICEPS muscle physiology”) OR SU (quadriceps OR quadriceps N3 function OR quadriceps N3 size OR quadriceps N3 power OR quadriceps N3 strength OR quadriceps N3 performance)) AND ((DE “MUSCLE strength” OR DE “MUSCLE weakness” OR DE “MUSCLE contraction” OR DE “MUSCULAR atrophy” OR DE “TORQUE”) OR SU (strength OR “muscle strength” OR “limb symmetry index” OR torque OR “peak torque” OR isokinetic OR dynamometr* OR power OR “muscle power” OR “rate of torque development” OR “RTD” OR “cross-sectional area” OR “CSA” OR “muscle size” OR volume OR girth OR contraction OR hypertroph* OR atroph* OR “fiber type” OR “fibre type”)) AND (SU (sex N3 differences OR sex N3 based OR sex N3 related OR sex N3 characteristics OR gender N3 differences OR gender N3 based OR gender N3 related OR male* OR female* OR man OR men OR Woman OR women))

**Results: 436**

## Appendix B

**Table A1:** Levels of certainty flow chart (GRADE).

Step 1: Establish Initial Level of Certainty	Step 2: Reasons for Considering Lowering or Raising Certainty		Step 3: Final Level of Certainty Rating
Study Design	Lower if:	Higher if:	Certainty in an Estimate of Effect Across Those Considerations
Randomized trials—High certainty	Risk of Bias	Large effect	High (formerly GRADE Level 1)
	Inconsistency	Dose response	Moderate (formerly GRADE Level 2)
	Indirectness	All plausible confounding and bias would reduce a demonstrated effect, OR would suggest a spurious effect if no effect was observed	Low (formerly GRADE Level 3)
Observational studies—Low certainty	Imprecision		Very Low (formerly GRADE Level 4)
	Publication bias		

## Appendix C

% Normative Values Peak Torque to Body Weight Graphs

## Appendix D

**Table A2:** Peak torque to body weight with normative reference values.

Study	Metric Class	Female (Mean ± SD)	Male (Mean ± SD)	Normative Values for Peak Torque to Bodyweight (Nm/kg)	% of Normative (Study Value/Normative Value) × 100
Otzel et al. [44]	Nm/kg, 60°/s	1.77 Nm/kg	2.45 Nm/kg	F = 2.27 Nm/kg M = 2.70 Nm/kg	F: 78% M: 90.7%
Otzel et al. [44]	Nm/kg, 60°/s	1.86 Nm/kg	2.55 Nm/kg	F = 2.27 Nm/kg M = 2.70 Nm/kg	F: 81.9% M: 96.4%
Otzel et al. [44]	Nm/kg, 180°/s	1.18 Nm/kg	1.86 Nm/kg	F = 2.27 M = 2.52 (60–65% band)	F: 52% M: 73.8%
Otzel et al. [44]	Nm/kg, 180°/s	1.28 Nm/kg	2.06 Nm/kg	F = 2.27 M = 2.52 (60–65% band)	F: 56.4% M: 81.7%
Zore et al. [46]	Nm/kg, 60°/s	1.79 ± 0.42 Nm/kg	2.00 ± 0.70 Nm/kg	F = 2.27 Nm/kg M = 2.70 Nm/kg	F: 78.9% M: 74.6%
Zore et al. [46]	Nm/kg, 60°/s	1.48 ± 0.44 Nm/kg	2.20 ± 0.50 Nm/kg	F = 2.27 Nm/kg M = 2.70 Nm/kg	F: 65.2% M: 81.5%
Zore et al. [46]	Nm/kg, 60°/s	1.38 ± 0.52 Nm/kg	2.10 ± 0.50 Nm/kg	F = 2.27 Nm/kg M = 2.70 Nm/kg	F: 60.8% M: 77.8%
Zore et al. [46]	Nm/kg, 60°/s	1.82 ± 0.57 Nm/kg	2.00 ± 0.70 Nm/kg	F = 2.27 Nm/kg M = 2.70 Nm/kg	F: 80.2% M: 74.1%
Rips et al. [48]	Nm/kg, 180°/s	1.18 ± 0.30 Nm/kg	1.45 ± 0.33 Nm/kg	F = 2.27 M = 2.52 (60–65% band)	F: 52.0% M: 57.5%
Rips et al. [48]	Nm/kg, 180°/s	1.14 ± 0.20 Nm/kg	1.57 ± 0.33 Nm/kg	F = 2.27 M = 2.52 (60–65% band)	F: 50% M: 62.3%
Rips et al. [48]	Nm/kg, 60°/s	1.88 ± 0.41 Nm/kg	2.14 ± 0.47 Nm/kg	F = 2.27 Nm/kg M = 2.70 Nm/kg	F: 82.8% M: 79.3%
Rips et al. [48]	Nm/kg, 60°/s	1.84 ± 0.34 Nm/kg	2.21 ± 0.51 Nm/kg	F = 2.27 Nm/kg M = 2.70 Nm/kg	F: 81.1% M: 81.9%

Note: Normative sources used in this review: (1) 60°/s and 180°/s Nm/kg—Van Melick et al. [19] (“Other Pivoting Sports” reference band). (2) Isometric torque, ~80–90° knee flexion—Barrué-Belou et al. [36] (35 men, 39 women, age 18–41, trained in ACL-injury-risk sports; isometric MVC at 80° knee flexion).

## Appendix E

**Table A3:** Heat flow diagram of outcome measures.

**Block A. Recovery-referenced (LSI peak torque, RFD-like).**							
**Outcome**	**Ebert ‘24**	**Kuenze ‘14**	**Otzel ‘15**	**San Jose ‘23**	**Zore ‘25**	**Yasuda ‘92**	**Rips ‘24**
LSI 90°/s @ 12 mo	+7.4						
LSI 90°/s @ 24 mo	+5.6						
MVIC concentric LSI 60°/s				+6.0			
MVIC concentric LSI 180°/s				+7.0			
Isometric MVC LSI				+6.0			
Isometric RTD LSI				+8.0			
Iso LSI 70° @ 3 mo						+18.0	
Iso LSI 70° @ 12 mo						+12.0	
Iso LSI 70° @ 4.5 y						+15.0	
Peak-torque LSI 60°/s (TSK > 37)							−8.0
Peak-torque LSI 180°/s (TSK > 37)							−8.0
Involved-limb deficit 60°/s			+3.0				
Involved-limb deficit 180°/s			−1.0				
**Block B. Absolute-capacity (Nm/kg, morphology).**							
**Outcome**	**Ebert ‘24**	**Kuenze ‘14**	**Otzel ‘15**	**San Jose ‘23**	**Zore ‘25**	**Yasuda ‘92**	**Rips ‘24**
Nm/kg MVIC pre-ex		+0.1					
Nm/kg MVIC post-ex		+0.3					
Nm/kg 60°/s involved			+0.7				
Nm/kg 60°/s uninvolved			+0.7				
Nm/kg 180°/s involved			+0.7				
Nm/kg 180°/s uninvolved			+0.8				
Nm/kg 60°/s (TSK > 37)							+0.3
Nm/kg 180°/s (TSK > 37)							+0.3
Thigh circumference—involved			+2.0				
Thigh circumference—control			+5.0				
**Block C. Normative-referenced (% of sex-specific normative capacity).**							
**Outcome**	**Ebert ‘24**	**Kuenze ‘14**	**Otzel ‘15**	**San Jose ‘23**	**Zore ‘25**	**Yasuda ‘92**	**Rips ‘24**
% Normative 60°/s involved			+12.7				
% Normative 60°/s uninvolved			+14.5				
% Normative 60°/s control cohort					−4.3		
% Normative 60°/s 2020 cohort					+16.3		
% Normative 60°/s 2021 cohort					+17.0		
% Normative 60°/s 2022 cohort					−6.1		
% Normative 60°/s (TSK > 37)							−3.5
% Normative 60°/s (TSK ≤ 37)							+0.8
% Normative 180°/s involved			+21.8				
% Normative 180°/s uninvolved			+25.3				
% Normative 180°/s (TSK > 37)							+5.5
% Normative 180°/s (TSK ≤ 37)							+12.3

Positive values favor males; negative values favor females; blank cells indicate the outcome was not reported for that study. Calculated as male delta − female delta for the quadriceps outcome value for each metric.
